# Computational Identification and Characterization of a Promiscuous T-Cell Epitope on the Extracellular Protein 85B of* Mycobacterium* spp. for Peptide-Based Subunit Vaccine Design

**DOI:** 10.1155/2017/4826030

**Published:** 2017-03-16

**Authors:** Md. Saddam Hossain, Abul Kalam Azad, Parveen Afroz Chowdhury, Mamoru Wakayama

**Affiliations:** ^1^Department of Biotechnology, Faculty of Life Sciences, Ritsumeikan University, Kusatsu, Shiga 525-8577, Japan; ^2^Department of Genetic Engineering & Biotechnology, Shahjalal University of Science and Technology, Sylhet 3114, Bangladesh; ^3^Department of Dermatology, Sylhet Women's Medical College, Sylhet 3114, Bangladesh

## Abstract

Tuberculosis (TB) is a reemerging disease that remains as a leading cause of morbidity and mortality in humans. To identify and characterize a T-cell epitope suitable for vaccine design, we have utilized the Vaxign server to assess all antigenic proteins of* Mycobacterium* spp. recorded to date in the Protegen database. We found that the extracellular protein 85B displayed the most robust antigenicity among the proteins identified. Computational tools for identifying T-cell epitopes predicted an epitope, 181-QQFIYAGSLSALLDP-195, that could bind to at least 13 major histocompatibility complexes, revealing the promiscuous nature of the epitope. Molecular docking simulation demonstrated that the epitope could bind to the binding groove of MHC II and MHC I molecules by several hydrogen bonds. Molecular docking analysis further revealed that the epitope had a distinctive binding pattern to all DRB1 and A and B series of MHC molecules and presented almost no polymorphism in its binding site. Moreover, using “Allele Frequency Database,” we checked the frequency of HLA alleles in the worldwide population and found a higher frequency of both class I and II HLA alleles in individuals living in TB-endemic regions. Our results indicate that the identified peptide might be a universal candidate to produce an efficient epitope-based vaccine for TB.

## 1. Introduction

Tuberculosis (TB) is a reemerging disease that remains a leading cause of morbidity and mortality in humans and represents a major public health problem in many countries [1]. Although the Bacille Calmette–Guérin (BCG) vaccine has been available for over 75 years, approximately one-third of the world's population was infected with* Mycobacterium tuberculosis* in 2011, resulting in an estimated 1.4 million people dying from the disease [2]. TB is caused by a group of phylogenetically related bacteria, the* M. tuberculosis* complex (MTBC) [3, 4], which is characterized by low overall genetic diversity and a largely clonal population structure [5–7]. MTBC has been classified into seven phylogenetic lineages, which are found in different geographic regions [6–12]. Although in most patients TB can be treated effectively with multidrug combinations of antibiotics, resistance to anti-TB drugs is increasing. TB treatment is undoubtedly inadequate for preventing disease transmission in highly endemic populations because an active TB patient usually infects approximately 10–15 people per year [2]. The current BCG vaccine has been extensively evaluated and has demonstrated variable protective efficacies ranging from 0% to 85% in different field trials [13]. The protective efficiency of BCG against pulmonary TB in adults, which represents the contagious form of this disease, is discrepant and incomplete, with BCG vaccination campaigns having only a modest impact on the incidence of pulmonary TB. One more issue of concern that compromises BCG's utility is that infants with HIV have an increased risk of developing disseminated BCG-osis [14]. This stresses the importance of developing TB vaccines that not only have a greater capability to provoke protective immunity against TB but also have a better safety outline than BCG [14, 15].

The first attempts to create better BCG vaccines were made by Tullius et al. [17] who developed a recombinant strain of* Mycobacterium* called rBCG30, which overexpressed antigen 85B (Ag85B). This vaccine resulted in improved protection against TB in guinea pigs and seemed to be immunogenic in humans [18]. A phase I clinical safety trial investigating rBCG30 has concluded but is currently on hold [19]. Since 2009, more than a dozen TB vaccine candidates have entered clinical trials, and many more are in the preclinical pipeline for testing in phase I clinical trials [20]. However, it should be noted that success in these studies and trials may not predict a vaccine's protective coverage on the diverse global stage [21]. Therefore, scientists are tasked with determining the global coverage of novel vaccine candidates through interdisciplinary preclinical approaches that integrate comparative genomics and bioinformatics [22, 23]. Though previous studies have suggested that* M. tuberculosis* has a comparatively stable genome in contrast with other bacteria [24, 25], genomic studies have exposed biologically significant variation among clinical strains [26]. Hebert and colleagues [27], for example, revealed considerable genetic variation in the* pepA* and* PPE18* genes of the clinical* M. tuberculosis* strain. Besides, the diversity of the most polymorphic regions of the human genome, the human leukocyte antigen (HLA) loci is thought to be a response to pathogen escape variants [28–30]. For this reason, to incorporate information of host genetic diversity in vaccine design, improvement, and assaying has been strongly emphasized [31].

Of the diverse vaccine candidates anticipated, subunit vaccines have received significant attention, particularly those composed of antigenic proteins Ag85B, ESAT-6, Ag85A, pepA, and PPE18 [32–34]. As these candidates move forward into clinical practices, it will be critically important to appraise their defensive potential as global vaccines via bioinformatics approaches to increase the understanding on the comparative genomics of the pathogen population and the immunomics of the host population. With the dawn of computers and informatics, novel approaches have been devised that focus on the development and applications of computational approaches to advance vaccine research and improve immunization programs. Epitope-based vaccines have attracted considerable concentration lately as a potential, inexpensive, and easy means of vaccine development for treating infectious disease [35, 36]. It was shown experimentally that a synthetic peptide can mimic the structure of epitopes and induce an immune response against the native proteins of bacteria causing Salmonellosis and* Chlamydia* infection [37, 38].

Surface and secreted proteins of any pathogen are mostly antigenic [39, 40] and are responsible for pathogenicity. The Ag85B is a secretory antigenic target and is highly conserved in MTBC [41–43]. The present study reports the identification and characterization of T-cell epitopes on Ag85B of* M. tuberculosis* and their interactions with the MHC molecules using different bioinformatics tools as well as molecular docking approaches. Furthermore, using the Allele Frequency Database, we investigated the frequency of the HLA alleles in individuals living in TB-endemic regions.

## 2. Materials and Methods

### 2.1. Identification of T-Cell Epitopes

Protegen (http://www.violinet.org/protegen/) [44], an antigen database and analysis system, was used to assess the antigenicity of Ag85B. For prediction of protective antigens and subunit vaccines, the VaxiJen (http://www.ddg-pharmfac.net/vaxijen/VaxiJen/VaxiJen.html) [45] system was utilized. Lastly, Vaxign (http://www.violinet.org/vaxign/) [36] was used for vaccine target prediction and analysis. The threshold value in VaxiJen was kept at 0.5. After confirmation of the highest antigenicity of Ag85B, all amino acid sequences of Ag85B of the pathogenic* Mycobacterium* spp. were retrieved from the protein database. The nonidentical sequences were subjected to multiple sequence alignment analysis with the T-Coffee server [46]. For identification of T-cell epitopes from Ag85B with strong MHC II binding affinity, artificial neural network based computer program NetMHCII 2.2 (http://www.cbs.dtu.dk/services/NetMHCII/) [47], motif matrices based SYFPEITHI (http://www.syfpeithi.de/) [48], and quantitative matrices based ProPred (http://www.imtech.res.in/raghava/propred/) [49] servers were used. To predict MHC I binding ability, the epitope sequences were subjected to the NetCTL 1.2 Server (http://www.cbs.dtu.dk/services/NetCTL/) [50]. Binding ability of the epitopes was analyzed against all 12 available MHC I HLA supertypes. Threshold values for “weight on C terminal cleavage,” “weight on TAP (transport associated antigen processing) transport efficiency,” and “epitope identification” were kept as 0.15, 0.05, and 0.75, respectively. Predicted MHC alleles that represented strong binding affinity with the epitope were studied in the “Allele Frequency Worldwide Populations database” [51] Additionally, 22 countries with high TB burden as identified by the World Health Organization (WHO) [2] were also analyzed for MHC alleles. Physicochemical properties of the epitope were analyzed with ProtParam computer program [52].

### 2.2. Retrieval of 3D Structure

The 3D structure of epitopes was built using PyMol molecule builder, as described previously [53]. There were no experimental structures available in the Protein Data Bank (PDB) (http://www.rcsb.org/pdb/home/home.do) [54] for the HLA-DRB1^*∗*^0701, HLA-DRB1^*∗*^0901, HLA-B39, and HLA-B58 molecules. The 3D structures of these four proteins were obtained using homology modeling with Molecular Operating Environment (MOE), as described previously [16]. The homology models of HLA-DRB1^*∗*^0701 [Uniprot:P13761], HLA-DRB1^*∗*^0901 [Uniprot:Q0R326], HLA-B39 [Uniprot:P30475], and HLA-B58 [Uniprot:P10319] were constructed based on the experimental structures of HLA-DR1 beta chain [PDB:1AQD-b], DRB1-1 beta chain [PDB:3PGD-b], HLA-B^*∗*^1402 alpha chain [PDB:3BXN-a], and HLA-B^*∗*^5701 alpha chain [PDB:3VRI-a], respectively. The amino acid sequence homology of HLA-DRB1^*∗*^0701, HLA-DRB1^*∗*^0901, HLA-B39, and HLA-B58 with HLA-DR1, DRB1-1, HLA-B^*∗*^1402, and HLA-B^*∗*^5701 is 89.31%, 87.90%, 97%, and 98%, correspondingly. To evaluate how much the structural models deviate from the template, the root-mean-square deviation (RMSD) value between the template and the homology model superposed was determined by the Internal Coordinate Mechanics method based computer program Molsoft Internal Coordinate Mechanics- (ICM-) pro 3.5 [55]. The stereochemical quality of the homology models was assessed by the PROCHECK program through the PDBsum server [56] (Table 1). The PDB ID for experimental structures of HLA-DRA [Uniprot:P01903], HLA-DRB1^*∗*^0101 [Uniprot:P04229], HLA-DRB1^*∗*^0404 [Uniprot:P13760], HLA-DPB1^*∗*^0101 [Uniprot:P04440], and HLA-DQB1^*∗*^0301 [Uniprot:P01920] were 1A6A-a, 1SJE-b, 1D5 M-b, 3LQZ-b, and 1JK8-b, respectively. MHC I HLA supertypes A2, A24, B8, B27, and B44 were retrieved from PDB. PDB ID for experimental structures of HLA supertype A2 [Uniprot:P01892], A24 [Uniprot:P05534], B8 [Uniprot:P30460], B27 [Uniprot:P03989], and B44 [Uniprot:P30481] were 1HHI-a, 3I6L-a, 1AGB-a, 10GT-a, and 1SYS-a, respectively.

### 2.3. Peptide Docking

The 3D structure of the Ag85B T-cell epitope built by the PyMol molecule builder was subjected to molecular docking simulation with MHC I and II molecules by Molsoft ICM-pro 3.5. The Ag85B T-cell epitope could bind to MHC II (HLA-DRB1^*∗*^0701, HLA-DRB1^*∗*^0101, HLA-DRB1^*∗*^0404, HLA-DRB1^*∗*^0901, HLA-DPB1^*∗*^0101, and HLA-DQB1^*∗*^0301) and MHC I (HLA-A2, HLA-A24, HLA-B27, HLA-B39, and HLA-B44) molecules. Molecular docking of the epitope was performed against the aforementioned 6 MHC II and 5 MHC I molecules. Ionizable group of cationic residues in neutral or mildly acidic compartment were transformed into the protonated states and ICM default partial atomic charges were set. ICM pocket finder macro was used for pocket selection, and after receptor positioning, the ICM method based molecular docking was done, as described previously [57]. The best docking orientation was selected based on binding free energy and hydrogen bond distance.

**Figure 1:**
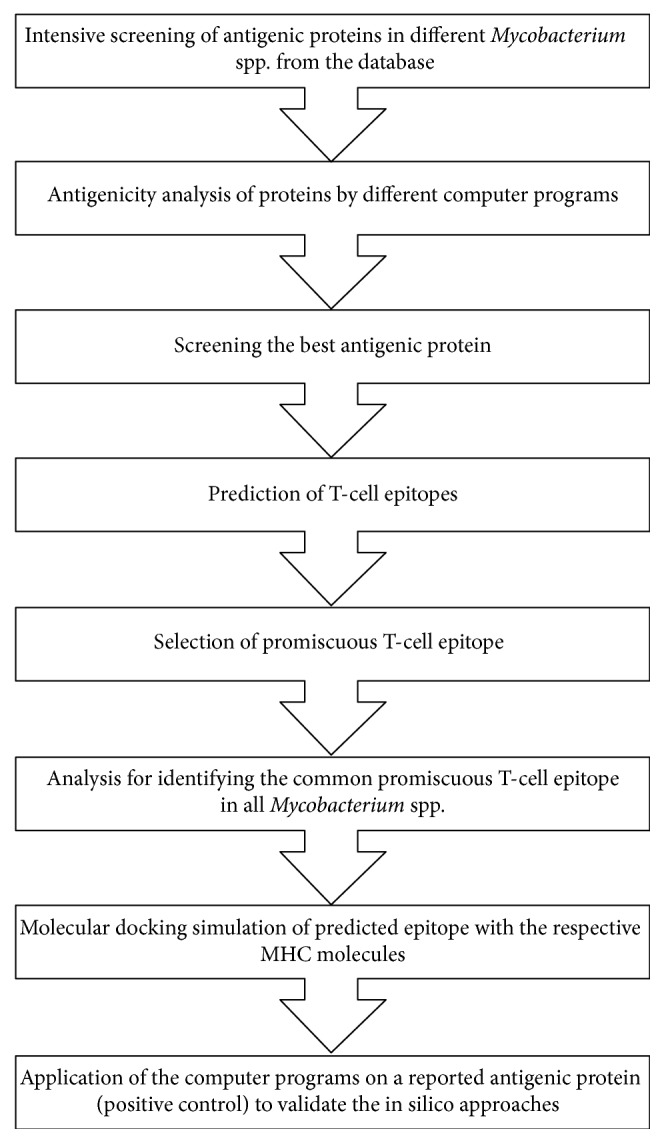
In silico approaches for T-cell epitope identification.

To validate the identification and characterization of the epitope with the computer-aided programs, the in silico approaches we used in this study were applied to identify the epitope on a positive control protein, an influenza viral antigen nucleoprotein (IvAgNP). Although IvAgNP is a viral antigenic protein, we used it as a positive control to cross check the in silico approaches used here and determine whether they can identify the epitopes previously reported [58]. The epitopes of IvAgNP were used as peptide vaccines [58, 59], and the epitope NP 380-ELRSRYWAIRTRSG-393 had been experimentally characterized [60, 61] and was known as an inducer of cellular response. A summary of in silico approaches used in this study is shown in Figure 1.

## 3. Results

### 3.1. Identification and Characterization of T-Cell Epitopes

Protegen analysis identified 24 experimentally verified protective antigens that had been reported to date in* M. tuberculosis*. Among the 24 antigenic proteins, 11 are extracellular, five are cytoplasmic, and one is a cell wall component. The localization of the remaining seven proteins is unknown. Five proteins, namely, Ag85B, Ag85A, ESAT-6, pepA, and PPE18, have been extensively studied and used as subunit vaccines in animal models [32–34]. Antigenicity analysis of these proteins with Vaxign (Table 2) showed that Ag85B, ESAT-6, and PEP18 had comparatively higher adhesion capability. However, it was concluded that it would be unwise to design a vaccine based on PEP18 due to polymorphism among clinical strains of* M. tuberculosis* [27]. The antigen probability was further analyzed with the VaxiJen server. The antigen probability for Ag85B was 0.5926, whereas that of ESAT-6 was 0.5657, indicating that Ag85B had higher antigenicity than ESAT-6. Therefore, Ag85B was considered for further analysis. Notably, the antigen probability of the positive control, IvAgNP, was only 0.5112. Ag85B was then subjected to the NetMHCII 2.2 web server and analyzed against 26 MHC II molecules available in the database. Ag85B showed strong binding capacity with 22 MHC II molecules (Table 3). The epitope with the strongest affinity was 181-QQFIYAGSLSALLDP-195. This epitope was found against HLA-DRB 1^*∗*^0101 with an affinity of 4.5 nM. Notably, an affinity below 50 nM in the NetMHCII 2.2 server is considered as strong binding. Upon further analysis, this epitope showed high affinity to a large number of MHC alleles (Table 4), revealing its promiscuous nature [57]. Four additional epitopes showed high affinity to HLA-DRB1^*∗*^0701, DPB1^*∗*^0201, DPB1^*∗*^0401, and DQB1^*∗*^0301 molecules compared to the epitope 181-QQFIYAGSLSALLDP-195 (Table 3). After being subjected to analysis through the Net MHCII 2.2 server, these epitopes showed an ability to bind only with the same MHC II molecule as shown in Table 3 with affinities below 50 nM, thereby showing a lack of promiscuous binding. The same approaches were applied to find the strongest promiscuous epitope on IvAgNP. The control IvAgNP also bound against 22 MHC II molecules (see Additional File 1, Table  S1 in Supplementary Material available online at https://doi.org/10.1155/2017/4826030). The most promiscuous epitope, 301-IDPFRLLQNSQVYSL-315, was found against HLA-DRB1^*∗*^0101 with an affinity of 3.4 nM (Additional File 2, Table  S2). A previously identified epitope, 380-ELRSRYWAIRTRSGG-393 was determined to be not promiscuous [58], though it did bind against HLA-DRB1^*∗*^0101 with a significant affinity of 9 nM (data not shown). The binding capability of Ag85B with MHC II molecules was further analyzed with ProPred and SYFPEITHI web servers. Each of these approaches predicted several epitopes that showed an ability to bind to many MHC II molecules. The 9-mer of the strongest predicted epitope mentioned above was identified as one of the top five epitopes in this analysis (Table 5). Data presented here were only for HLA-DRB1^*∗*^0701.

To predict MHC I binding ability, epitope sequences were subjected to the NetCTL 1.2 Server. The binding ability of the epitopes was analyzed against all 12 available MHC I HLA supertypes. Analysis showed that the 9-mer of the predicted epitope, 181-QQFIYAGSLSALLDP-195, could bind significantly with five MHC I HLA supertypes, namely, A2, A24, B27, B44, and B39. The 9-mer epitope 181-QQFIYAGSL-189 could bind to B27 and B44 MHC I HLA supertypes. However, the 9-mer epitope 184-IYAGSLSAL-192 could bind to both HLA-A24 and B39 supertypes. Likewise, HLA-A2 supertypes could bind to the epitope 183-FIYAGSLSA-191. Analysis with the NetCTL 1.2 server revealed that the 9-mer of the positive control epitope, 380-ELRSRYWAIRTRSG-393 of IvAgNP, could bind to only two MHC I HLA supertypes, namely, B8 and B27. However, the strongest epitope, 301-IDPFRLLQNSQVYSL-315, could bind significantly against five MHC I allele supertypes, namely, A3, A2, B27, B39, and B58.

**Figure 2:**
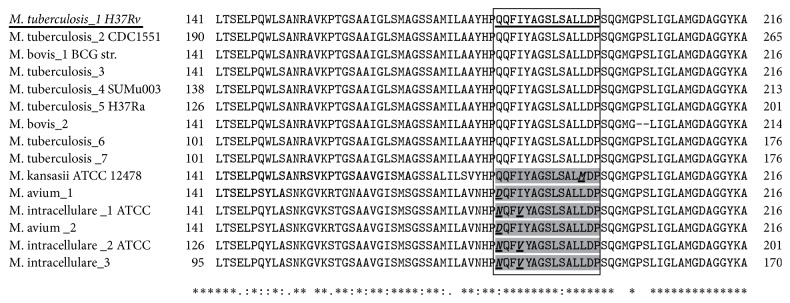
Multiple alignment of protein sequences of Ag85B from* Mycobacterium* spp. Accession numbers of Ag85B from* M. tuberculosis* were NP_216402 (*M. tuberculosis*_1 H37Rv), NP_336393 (*M. tuberculosis*_2 CDC1551), 1F0PA (*M. tuberculosis*_3), ZP_07422952 (*M. tuberculosis*_4 SUMu003), ZP_02552646 (*M. tuberculosis*_5 H37Ra), 1F0N_A (*M. tuberculosis*_6), and AAO62005 (*M. tuberculosis*_7). Accession numbers of Ag85B from* M. bovis* were YP_978013 (*M. bovis*_1 BCG str.) and AAA25359 (*M. bovis*_2). Accession numbers of Ag85B from* M. intracellulare* were YP_005338175 (*M. intracellulare* _1 ATCC), BAA03981 (*M. intracellulare*_2 ATCC), and BAA03243 (*M. intracellulare*_3). Accession numbers of Ag85B from* M. avium* were Q06947 (*M. avium*_1) and AAM21939 (*M. avium*_2), and those of Ag85B from* M. kansasii* were ZP_04749192 (*M. kansasii* ATCC 12478). The best epitope based on conservation and NetMHCII 2.2 analysis is shown in the box. The epitope sequence from* M. tuberculosis*_1 H37Rv is underlined. Nonidentical epitope sequences are shaded and the amino acid residues for which the epitope varies are bold, italic, and underlined.

To predict the universal effectiveness of the epitope, we retrieved all 150 Ag85B sequences (Additional File 3, Table  S3) of pathogenic* Mycobacterium* spp. from the protein database and found 15 nonidentical Ag85B sequences. These nonidentical sequences were then subjected to the T-Coffee server for multiple sequence alignment to determine the identity and the universality of the predicted epitopes among pathogenic* Mycobacterium* spp. (Figure 2). This analysis revealed that the percentage of identity of the predicted strongest epitope was 100, 93, and ~87 in 9, 3, and 3* Mycobacterium* spp., respectively. These results revealed that the predicted strongest epitope had at least 90% of amino acid identity in all* Mycobacterium* spp. indicating the universality of the epitope. Likewise, we retrieved 1300 IvAgNP sequences (data not shown) of pathogenic influenza virus and 73 nonidentical sequences were aligned to find the conservation of the control epitope and the strongest promiscuous epitope (Additional File 4, Figure  S1). Figure  S1 shows the percentage of identity in amino acid level of the predicted strongest epitope was 100, 93, and 86 in 6, 42, and 25 influenza A viruses, respectively. The level of identity was 86.75% in all influenza A virus.

Physicochemical properties of the epitope include a molecular weight of 1622.8 Da with 230 atoms (C_75_H_115_N_17_O_23_). Analysis with the ProtParam computer program revealed that the instability index was 32.93. This result indicates a high stability of the epitope since a ProtParam instability index smaller than 40 is considered to be stable. However, the ProtParam instability index of the positive control epitope and the predicted strongest promiscuous epitope of IvAgNP was 76.04 and 46.69, respectively, indicating that these epitopes are unstable.

### 3.2. Docking of the Predicted T-Cell Epitope to MHC Molecules

**Figure 3:**
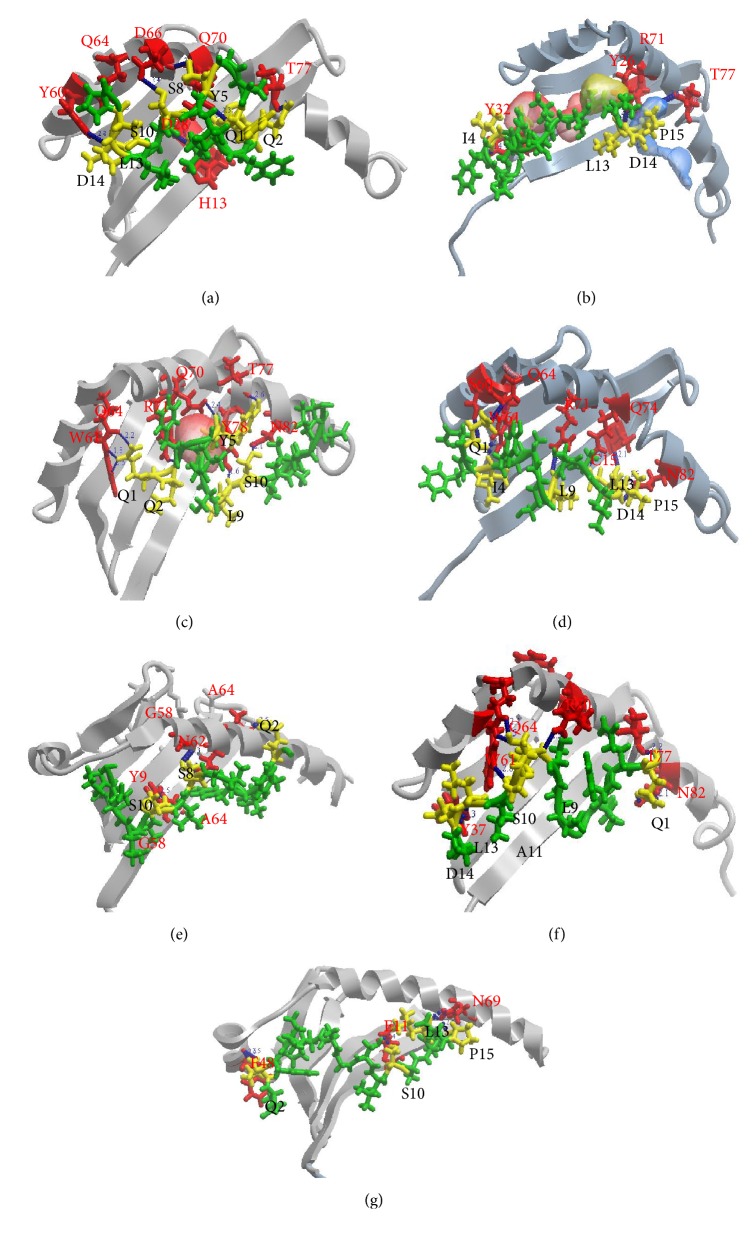
Docking of the epitope with HLA-DRB1^*∗*^0101 (a), HLA-DRB1^*∗*^0901 (b), HLA-DRB1^*∗*^0404 (c), HLA-DRB1^*∗*^0701 (d), HLA-DPB1^*∗*^0101 (e), HLA-DQB1^*∗*^0301 (f), and HLA-DRA (g). The 3D structure of the Ag85B T-cell epitope was made in PyMol molecule builder. Docking of receptor MHC with epitope was performed in Molsoft ICM-pro 3.5. All 3D structures of HLA proteins were obtained from the PDB server unless noted. Homology models of HLA-DRB1^*∗*^0701 and HLA-DRB1^*∗*^0901 were prepared in MOE as described by Azad et al. [16]. MHC II structures are shown in gray ribbon and the amino acid residues involved in the hydrogen bonding interaction are shown as red sticks and labeled red. The epitope is shown as green ribbon and the amino acid residues involved in the hydrogen bonding network are shown as yellow sticks and labeled black. MHC II binding pockets are shown as an electrostatic sphere. Hydrogen bonds are displayed as blue spheres and the hydrogen bonding distances are labeled blue. Hydrogen bonding interaction between the amino acid residues of the epitope and those of MHC II are detailed in Additional File 5, Table  S4.

Molecular docking was performed to analyze the interaction of the epitope with MHC molecules. Docking showed that the protrusion of the side chains of the epitope bound in cavities within the groove of MHC II HLA-DRB1^*∗*^0101 through nine hydrogen bonds (Figure 3(a); Additional File 5, Table  S4). Docking simulation revealed that the binding energy between the epitope and HLA-DRB1^*∗*^0101 was −181.93 kcal/mol. A binding energy less than −32 kcal/mol is generally considered a good score in the ICM method [55]. Molecular docking of the epitope was further performed with DRB1^*∗*^0901, DRB1^*∗*^0404, DRB1^*∗*^O701, DPB1^*∗*^0101, and DQB1^*∗*^0301 (Figures 3(b)–3(f)). Docking analysis showed that the epitope side chains bound in the cavities within the grooves of HLA-DRB1^*∗*^0404, DRB1^*∗*^O701, DRB1^*∗*^0901, DPB1^*∗*^0101, and DQB1^*∗*^0301 through 9, 8, 6, 5, and 9 hydrogen bonds, respectively, and the binding energy of the epitope with these MHC II molecules were −162.42, −179.42, −178.35, −159.39, and −202.60 kcal/mol, correspondingly (Additional File 5, Table  S4). As HLA-DRA does not have polymorphisms in the peptide binding region, we performed molecular docking simulation between the epitope and HLA-DRA (Figure 3(g)). Docking analysis revealed that the epitope bound to the groove by six hydrogen bonds with the binding energy of −204.17 kcal/mol. Docking analysis of the predicted strongest epitope, 301-IDPFRLLQNSQVYSL-315, on the positive control protein showed that the epitope side chains bound in the cavities within the grooves of HLA-DRB1^*∗*^0101, DRB1^*∗*^0901, DRB1^*∗*^0404, and DRB1^*∗*^0701 through 9, 9, 7, and 8 hydrogen bonds, respectively, and the binding energy of the epitope with these MHC II molecules were −194.87, −211.17, −189.62, and −185.42 kcal/mol, correspondingly (Additional File 6, Figure  S2; Additional File 7, Table  S5). However, docking analysis showed that the epitope 380-ELRSRYWAIRTRSG-393 in IvAgNP bound within the grooves of only HLA-DRB1^*∗*^0101 through 10 hydrogen bonds with a binding energy of −191.05 kcal/mol (data not shown).

**Figure 4:**
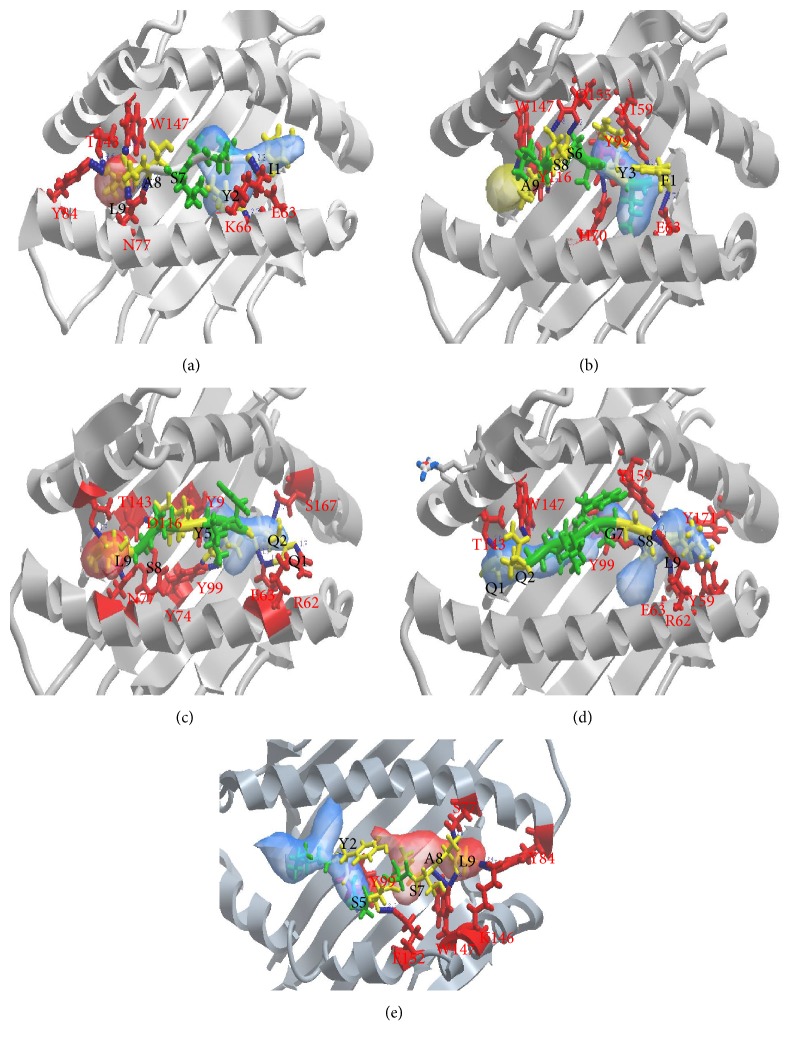
Docking of the epitope with HLA-A24 (a), HLA-A2 (b), HLA-B44 (c), HLA-B27 (d), and HLA-B39 (e). Building of the 3D structure of the Ag85B T-cell epitope and 3D models of HLA-B39 and the docking of receptor MHC with the epitope were performed as mentioned in Figure 3. MHC I structures are shown in gray ribbon and the amino acid residues involved in the H-bonding interaction are shown as red sticks and labeled red. The epitope is shown as green ribbon and the amino acid residues involved in the H-bonding network are shown as yellow sticks and labeled black. MHC I binding pockets are shown as electrostatic sphere. H-bonds are displayed as blue spheres and the H-bonding distances are labeled blue. H-bonding interaction between the amino acid residues of the epitope and those of the MHC I are detailed in Additional File 8, Table  S6.

Docking of the 9-mers of the predicted epitope was performed with MHC I molecules. Docking analysis showed that the 9-mer epitope 183-FIYAGSLSA-191 bound to HLA-A2 through eight hydrogen bonds with a binding energy of −162.01 kcal/mol (Figure 4; Additional File 8, Table  S6). The epitope 184-IYAGSLSAL-192 bound in the cavities within the groove of HLA-A24 and B39 through seven hydrogen bonds with a binding energy of −185.45 and −175.42 kcal/mol, respectively (Figure 4; Additional File 8, Table  S6). The 9-mer epitope 181-QQFIYAGSL-189 bound to HLA-B27 and HLA-B44 through 11 hydrogen bonds with a binding energy of −193.62 and −200.13 kcal/mol, respectively (Figure 4; Additional File 8, Table  S6). Docking analysis also revealed that the 9-mers of the epitopes in IvAgNP bound to MHC I supertypes with a significant number of hydrogen bonds and binding energy (Additional Files 9 and 11, Figures  S3, S4; Additional Files 10 and 12, Tables  S7, S8).

### 3.3. Receptor Binding Sites for the Epitope 181-QQFIYAGSLSALLDP-195

**Figure 5:**
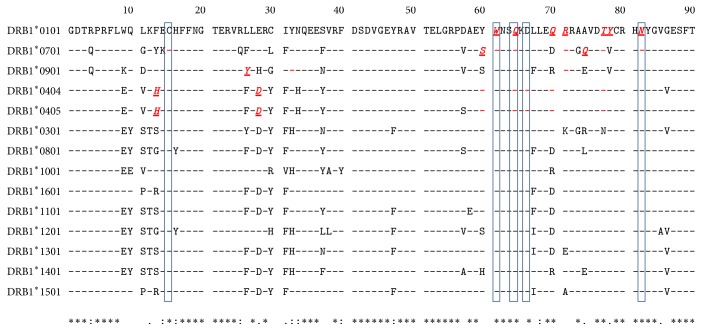
Multiple sequence alignment of amino acid sequences of HLA-DRB1 series. The T-Coffee server was used for multiple alignment of the N-terminal 90 amino acid residues of the HLA-DRB alleles. The HLA-DRB1^*∗*^0101 sequence was chosen as reference. Identities of the residues are illustrated by dashes. Positions 15(C), 61(W), 64(Q), 66(D), and 82(N) are indicated as boxes to present no polymorphic binding site in the MHC protein. Binding residues are labeled red, bold, italic, and underlined. Identical binding residues in MHC proteins are indicated as red dashes.

The MHC II binding site is formed by 35 amino acids from the N-terminal 80 and 90 residues of the HLA-DRA and HLA-DRB, respectively [52, 55, 62]. As the HLA-DRA exhibits no binding site polymorphisms [63], we used only the HLA-DRB1 chains for analysis. Our analysis revealed that DRB1 chains contain 29 polymorphic amino acids in the binding sites. The polymorphic positions are 4, 9, 10, 11, 12, 13, 14, 16, 26, 28, 30, 31, 32, 33, 37, 38, 40, 47, 57, 60, 67, 70, 71, 73, 74, 77, 78, 85, and 86 (Figure 5). Docking analysis of the receptor position showed that the epitope bound to 15 positions of HLA-DRB1 alleles. Among them, positions 15(C), 61(W), 64(Q), 66(D), and 82(N) presented no polymorphism in the receptor binding site. However, only one residue among the positions 28(D), 32(Y), 60(Y), 77(T), and 78(Y) of the DRB1 chain was found to be substituted. We also analyzed interaction sites (residues 1–179) of MHC I molecules and found that the peptide interacted at 17 positions of HLA-A and B proteins (Additional File 13, Figure  S5). Among them, residues at positions 59(Y), 84(Y), 99(Y), 143(T), 147(W), 155(Q), and 159(Y) showed no polymorphism.

## 4. Discussion

Peptide or epitope-based vaccines are specific and induce immunity against the selected epitope(s). Due to this specificity, these vaccines can avoid immune responses induced by unfavorable epitopes of the antigen, thereby preventing complications [64]. Along with increased safety, peptide-based vaccines give the opportunity to engineer the epitope to increase its universality, potency, and breadth [65]. In addition, epitope-based vaccines have been shown to be successful in preventing infectious diseases like influenza [66]. Small epitopes (usually 8–15 amino acids in length) can be delivered easily by any of the following devices such as peptide-adjuvant conjugates (lipid-core peptides), peptide amphiphiles, lipid-based synthetic vesicles, endogenous exosomes containing peptide-loaded MHC molecules, noninfectious virus-like particles displaying recombinant epitopes, surface-conjugated peptides, and solid-core nanobeads with conjugated peptides [67]. The effectiveness of a peptide-based vaccine is greatly dependent on the exact identification of the immunogenic epitopes that bestow protection. In this study, different computer-aided bioinformatics tools have been used to identify the potentially promiscuous epitope 181-QQFIYAGSLSALLDP-195 from Ag85B, which may induce T-cell-mediated immunity against* Mycobacterium* spp. In the Immune Epitope Database, the Ag85B epitopes are recognized as immunodominant peptides that may react with human T-cells [68]. Computational tools used in our analysis have been validated by applying them on a reported IvAgNP epitope that was used experimentally as a vaccine following characterization of its epitope, 380-ELRSRYWAIRTRSG-393. The epitope of IvAgNP was associated with CD8+ cell-based immunity [60, 61]. Through analysis by these tools, we found the same HLA allele that was previously characterized. However, the epitope 380-ELRSRYWAIRTRSG-393 did not possess high potentiality to induce both types of cell-based immunity. Through our analysis on IvAgNP, we identified several limitations of its epitope, including (i) lack of high conservation in almost all pathogenic influenza viruses, (ii) absence of a promiscuous nature, and (iii) a ProtParam instability index greater than 40 that indicates a significantly reduced half-life. These limitations may suggest why this epitope was only investigated at the experimental level, never reaching successful epitope-based vaccination. However, the strongest epitope identified in our study, 181-QQFIYAGSLSALLDP-195 on Ag85B, had overcome such limitations.

Analysis with Vaxign revealed that Ag85B was more antigenic than other immunogenic mycobacterial proteins. Results of analyses with NetMHCII 2.2, SYFPEITHI, ProPred, and NetCTL 1.2 servers confirmed that the predicted epitope from Ag85B of* M. tuberculosis *could bind to several MHC II and MHC I molecules and thus revealed its promiscuous nature [52, 57]. Epitope promiscuity is usually assessed in the generation of epitope-based vaccines [69–71]. In addition, promiscuously binding antigenic epitopes are considered to act against a vast range of host immune systems.

It is important to recognize the inherent limitations of epitope prediction programs. No epitope prediction program is absolutely accurate, and there is substantial discrepancy among the epitopes predicted by each program [72, 73]. We increased the accuracy of our analysis by using multiple prediction programs for each DRB allele that have undergone extensive validation [47, 72, 73]. Fortunately, there was agreement among programs. We focused more on the class II MHC DRB1 alleles because of the comparative abundance of data on epitopes binding to DR alleles. It has been documented for TB that more than 90% of class II* M. tuberculosis* epitopes bind to DR antigens [74]. In addition, the* DRB1* gene was extensively studied because the expression level of DRB1 proteins is typically fivefold greater than that of* DRB3, DRB4*, or* DRB5* gene [75].

NetCTL 1.2 is an integrative approach to CTL epitope prediction that predicts MHC I binding, TAP transport efficiency, and proteasomal cleavage [50, 62]. Therefore, the epitope's ability to induce cytotoxic T-cell (CD8+), and natural killer cell (inhibitory receptor), mediated immunity might be satisfactory as it can bind to five MHC I HLA (two A and three B) supertypes. Immunity to TB in humans, primates, and mice depends on T lymphocytes [76, 77]. So the treatment of TB actually requires cell-mediated immunity that includes both CD4+ and CD8+ T-cells [4, 18, 19, 78]. MHC II molecules interact with CD4+ T helper cells that stimulate macrophages to kill phagocytosed pathogens. Since MTBC inhibits macrophages, CD4+ T-cell-mediated immunity is particularly important for preventing and clearing MTBC infection [18].

Little is known about the amino acid variation of Ag85B proteins in* Mycobacterium* spp. If the proposed epitope sequence is highly variable among the Ag85B, the protective efficacy of Ag85B as an epitope-based vaccine might be compromised on the global stage. Our investigation showed that the proposed epitope had 100% identity among MTBC and 87–93% identity among the remaining 40% of* Mycobacterium* spp., defining it as a universal epitope (Figure 2). A seminal observation of 22 genomes of MTBC reveals that most of the experimentally identified human T-cell epitopes are hyperconserved on the evolutionary scale [4]. These findings provide evidence that the relationship between MTBC and its human host actually may differ from a classical evolutionary arms-race and informs the development of new approaches to control TB. However, recently, Copin et al. described computationally and experimentally that highly conserved MTB T-cell epitopes in fact contribute to pathology [30]. Therefore, it is noteworthy that a vaccine encoding conserved promiscuous epitopes not only shows protective immunity against wide range of pathogenic strains but also induces broad T-cell responses [79–81].

Recently, the incidence of Nontuberculous Mycobacterial (NTM) infections has surpassed the incidence of TB infections in developed countries [82–86]. Although infection may occur virtually in any organ, pulmonary infections are most common [87–89] with* M. avium* and* M. kansasii* as the causative agents of lung disease [82, 84, 90]. Difficulty eradicating NTM and its high rate of reoccurrence have made it complicated to make clinical decisions for treatment. The proposed epitope in our study had 87–93% identity among* M. avium, M. kansasii*, and* M. intracellulare*. Therefore, this vaccine candidate would potentially target the diversity of* Mycobacterium* spp. worldwide. Multiple alignment of the Ag85B protein sequence showed that glutamine (Q181 in* M. tuberculosis*-1 H37Rv) present in 10 Ag85B sequences is substituted by aspartic acid and asparagine in* M. avium* and* M. intracellulare*, respectively. Isoleucine (I184 in* M. tuberculosis*-1 H37Rv) present in 12 Ag85B sequences is substituted by valine in* M. intracellulare*. Leucine (L192 in* M. tuberculosis*-1 H37Rv) present in 14 Ag85B sequences is substituted by methionine in* M. kansasii*. Glutamine has strongly similar properties (roughly the same size and the same hydropathy) as aspartic acid and asparagine, while isoleucine is similar to valine [91]. Therefore, the substituted amino acid residues in the predicted epitope of Ag85B in only ~33% of* Mycobacterium* spp. may not alter the properties of the epitope to change its universality. ProtParam analysis of the epitope with the substituted amino acid(s) to mimic the polymorphism in* M. avium*,* M. intracellulare*, and* M. kansasii* showed almost the same half-life and instability index as that of the predicted epitope (data not shown). Thus, comparative proteomics of the pathogen population from a greater range of geographic origins stands as an additional useful tool for preclinical evaluation of new vaccine targets.

The Ag85B T-cell epitope formed a number of significant hydrogen bonds with the binding groove of each of the MHC II and MHC I molecules (Figures 3 and 4; Additional Files 5 and 8, Tables  S4, S6). Hydrogen bonding distance less than 3 Å is usually considered biologically significant. The binding energy of the Ag85B T-cell epitope with each of the MHC I and II molecules is also noteworthy since a binding energy below −32 kcal/mol is biologically significant [55]. The epitope has the ability to bind to both the residues inside the groove as well as outside the binding groove (flanking residues) of the MHC molecules. This is considered as a principal determinant of epitope binding affinity [53, 57, 92–97].

It is necessary to incorporate information on host and pathogen genetic diversity in vaccine design, development, and testing. The assortment of the HLA and other genes involved in the immune system results in variable vaccine efficacy in different individuals even in the absence of pathogen variation [98]. Our analysis of the “Allele Frequency Database” revealed that the HLA alleles to which the predicted epitope bound were commonly found in TB-endemic regions. Among them, the DRB1 alleles have the highest frequency (~95%) in the TB burden countries. This result stresses the notion that the proposed epitope may confer an enhanced immunity to the patients of certain endemic region of the world. Furthermore, it might be a better candidate as an epitope-based vaccine because our data showed that the epitope had preference for both CD4+ and CD8+ T-cell-based immunity. Additionally, the binding residues of MHC had almost no polymorphism in all MHC DRB1 and MHC A and B series of proteins. Therefore, the predicted epitope would induce immunity against almost all class I and class II alleles and may have great implication for an epitope-based vaccine with the potential to impact the global population.

## 5. Conclusion

Here, we report (i) high percentage of identity of the epitope in all Ag85B sequences, (ii) adhesin capability and antigen probability of Ag85B from* M. tuberculosis*, (iii) strong binding affinity of the epitope with MHC I and II molecules, (iv) physicochemical properties of the epitope, (v) molecular docking simulation, and (vi) coverage of HLA alleles in TB-endemic countries, which supports the notion that the proposed epitope might be a novel universal efficient epitope to produce peptide or epitope-based vaccines against TB. While biochemical analysis remains necessary to validate the interaction between the epitope and the MHC molecules through elucidation of immunity induction, most of the approaches have been validated by applying them on a positive control protein, IvAgNP.

## Supplementary Material

Additional file 1-Table S1: Binding of epitopes of IvAgNP from Influenza virus against MHC II alleles.
Additional file 2-Table S2: Binding affinity of predicted strongest epitope 301-IDPFRLLQNSQVYSL-315 from IvAgNP to MHC alleles.
Additional file 3-Table S3: Accession numbers of antigenic Ag85B sequences of *Mycobacterium *pathogenic strains found in protein database.
Additional file 4-Figure S1: Multiple alignment of protein sequences of IvAgNP from Influenza virus.
Additional file 5-Table S4: Hydrogen Bonds and binding statistics of epitope 181-QQFIYAGSLSALLDP-195 from Ag85B with MHC II complexes.
Additional file 6-Figure S2: Docking of predicted strongest epitope 301-IDPFRLLQNSQVYSL-315 from IvAgNP with HLA-DRB1∗0101 (A), DRB1∗0901 (B), DRB1∗0404 (C) and DRB1∗0701 (D).
Additional file 7-Table S5: Hydrogen Bonds and binding statistics of predicted strongest epitope 301-IDPFRLLQNSQVYSL-315 from IvAgNP with MHC II complexes.
Additional file 8-Table S6: Hydrogen Bonds and binding statistics of epitope 181-QQFIYAGSLSALLDP-195 from Ag85B with MHC I complexes.
Additional file 9-Figure S3: Docking of control epitope 380-ELRSRYWAIRTRSG-393 from IvAgNP with HLA-B8 (A) and B27 (B).
Additional file 10-Table S7: Hydrogen Bonds and binding statistics of control epitope 380-ELRSRYWAIRTRSG-393 from IvAgNP with MHC I complexes.
Additional file 11-Figure S4: Docking of predicted strongest epitope 301-IDPFRLLQNSQVYSL-315 from IvAgNP with HLA-A2 (A), A3 (B), B27 (C), B39 (D) and B58 (E).
Additional file 12-Table S8: Hydrogen Bonds and binding statistics of predicted strongest epitope 301-IDPFRLLQNSQVYSL-315 from IvAgNP with MHC I complexes.
Additional file 13-Figure S5: Multiple alignments of amino acid sequences of HLA-A and HLA-B series.

## Figures and Tables

**Table 1:** Ramachandran plot statistics computed with PROCHECK program.

Plot statistics	DRB1^*∗*^0701	DRB1^*∗*^0901	HLA-B39	HLA-B58
% residues in favourable regions	89.5	89.2	92.8	92.0
% residues in additional residue regions	9.9	10.8	6.6	7.7
% residues in generously regions	0.0	0.0	0.6	0.3
% residues in disallowed regions	0.6	0.0	0.0	0.0
% of nonproline and nonglycine residues	100.00	100.00	100.00	100.00

**Table 2:** Comparison of antigenicity of antigenic proteins of *Mycobacterium *spp. analyzed with Vaxign computer program.

Protective antigens	Accession number	Vaxign analysis		
		Localization probability	Adhesin probability	Number of similar human proteins
Ag85B	NP_216402.1	Periplasmic (Prob. = 0.984)	0.618	0
Ag85A	AAO62004.1	Periplasmic (Prob. = 0.984)	0.558	0
ESAT-6	YP_178023.1	Unknown (Prob. = 0.2)	0.659	0
pepA	NP_214639.1	Periplasmic (Prob. = 0.976)	0.456	4
PPE18	YP_177795.1	Cytoplasmic membrane (Prob. = 0.973)	0.650	0
IvAgNP^*∗*^	P03466	Periplasmic (Prob. = 0.983)	0.076	0

^*∗*^Positive control.

**Table 3:** Binding of epitopes of Ag85B from *M. tuberculosis *against MHC II molecules^a^.

MHC II molecule	Strongest epitope				Number of strong binding epitopes	Number of weak binding epitopes
	Start position	Full peptide (15 mer)	Core peptide (9 mer)	Affinity (nM)		
*HLA-DRB1* ^*∗*^ *0101*	*181*	*QQFIYAGSLSALLDP*	*FIYAGSLSA*	*4.5*	*106*	*105*
HLA-DRB1^*∗*^0301	269	LENFVRSSNLKFQDA	FVRSSNLKF	17.1	6	26
HLA-DRB1^*∗*^0401	46	LPVEYLQVPSPSMGR	YLQVPSPSM	18.1	9	95
HLA-DRB1^*∗*^0404	46	LPVEYLQVPSPSMGR	VEYLQVPSP	22.3	10	99
HLA-DRB1^*∗*^0405	180	PQQFIYAGSLSALLD	FIYAGSLSA	18.5	18	65
HLA-DRB1^*∗*^0701	179	HPQQFIYAGSLSALL	FIYAGSLSA	4.3	49	36
HLA-DRB1^*∗*^0901	181	QQFIYAGSLSALLDP	YAGSLSALL	9.2	25	91
HLA-DRB1^*∗*^1101	5	SRKIRAWGRRLMIGT	IRAWGRRLM	17.3	12	52
HLA-DRB1^*∗*^1302	269	LENFVRSSNLKFQDA	FVRSSNLKF	13.5	8	35
HLA-DRB1^*∗*^1501	3	DVSRKIRAWGRRLMI	KIRAWGRRL	13.9	9	90
HLA-DRB3^*∗*^0101	74	PAVYLLDGLRAQDDY	YLLDGLRAQ	18	13	40
HLA-DRB4^*∗*^0101	59	GRDIKVQFQSGGNNS	IKVQFQSGG	13.9	6	48
HLA-DRB5^*∗*^0101	269	LENFVRSSNLKFQDA	FVRSSNLKF	9.2	21	67
HLA-DPA1^*∗*^0103-DPB1^*∗*^0401	132	CQTYKWETFLTSELP	YKWETFLTS	3.9	12	31
HLA-DPA1^*∗*^0103-DPB1^*∗*^0201	131	GCQTYKWETFLTSEL	YKWETFLTS	2.3	17	55
HLA-DPA1^*∗*^0201-DPB1^*∗*^0101	132	CQTYKWETFLTSELP	YKWETFLTS	7.7	17	40
HLA-DPA1^*∗*^0201-DPB1^*∗*^0501	132	CQTYKWETFLTSELP	YKWETFLTS	39.7	2	18
HLA-DPA1^*∗*^0301-DPB1^*∗*^0402	132	CQTYKWETFLTSELP	YKWETFLTS	21.7	10	44
HLA-DQA1^*∗*^0101-DQB1^*∗*^0501	114	GQSSFYSDWYSPACG	QSSFYSDWY	19.4	5	21
HLA-DQA1^*∗*^0102-DQB1^*∗*^0602	165	LSMAGSSAMILAAYH	GSSAMILAA	7.1	24	86
HLA-DQA1^*∗*^0501-DQB1^*∗*^0201	259	LGGANIPAEFLENFV	IPAEFLENF	34.5	3	24
HLA-DQA1^*∗*^0501-DQB1^*∗*^0301	27	GLVGLAGGAATAGAF	GLAGGAATA	3.7	82	94

^a^Binding of epitopes of Ag85B from *M. tuberculosis *was predicted using NetMHCII 2.2 server in accordance with the instruction of the server. 22 MHC II molecules against which bindings were strong are shown. Binding affinity below 50 nM is considered strong. The strongest epitope bound to HLA-DRB1^*∗*^0101 MHC II molecule is italicized.

**Table 4:** Binding of 181-QQFIYAGSLSALLDP-195 epitope of Ag85B to respective allele predicted by NetMHCII 2.2 servers^a^.

MHC II alleles	Promiscuous peptide sequence		Affinity (nM)
	Full peptide (15 mer)	Core peptide (9 mer)	
HLA-DRB1^*∗*^0101	QQFIYAGSLSALLDP	FIYAGSLSA	4.5
HLA-DRB1^*∗*^0404	QQFIYAGSLSALLDP	FIYAGSLSA	49.1
HLA-DRB1^*∗*^0405	QQFIYAGSLSALLDP	FIYAGSLSA	20.7
HLA-DRB1^*∗*^0701	QQFIYAGSLSALLDP	FIYAGSLSA	6.1
HLA-DRB1^*∗*^0901	QQFIYAGSLSALLDP	FIYAGSLSA	9.2
HLA-DRB5^*∗*^0101	QQFIYAGSLSALLDP	FIYAGSLSA	33.7
HLA-DPA1^*∗*^0201-DPB1^*∗*^0101	QQFIYAGSLSALLDP	FIYAGSLSA	38.1
HLA-DQA1^*∗*^0501-DQB1^*∗*^0301	QQFIYAGSLSALLDP	FIYAGSLSA	5.7

^a^Binding affinity below 50 nM is considered as strong immunogenicity.

**Table 5:** Binding of epitopes of Ag85B from *M. tuberculosis *against HLA-DRB1^*∗*^0701 molecules^a^.

Epitope rank	Start position	Sequence	Score
*Predicted with ProPed*			
1	271	FVRSSNLKF	8
2	166	MAGSSAMIL	5.9
3	154	VKPTGSAAI	5.8
4	184	*YAGSLSALL*	5.7
5	101	YQSGLSIVM	5.3

*Predicted with SYFPEITHI*			
1	152	NRAVKPTGSAAIGLS	32
2	182	QFI*YAGSLSALL*DS	32
3	162	AIGLSMAGSSAMILA	30
4	180	PQQFIYAGSLSALLD	28
5	217	ADMWGPSSDPAWERN	28

^a^Binding of epitopes of Ag85B from *Mycobacterium* spp. was predicted by analyzing with ProPred and SYFPEITHI servers. The higher the score of an epitope, the greater the probability of binding to a given MHC molecule. The core sequence (9-mer) of the epitope 181-QQFIYAGSLSALLDP-195 is italicized. Five epitopes with the top scores are shown.
