# Supplementary material for: Computational Identification and Characterization of a Promiscuous T-Cell Epitope on the Extracellular Protein 85B of Mycobacterium spp. for Peptide-Based Subunit Vaccine Design
Source: Biomed Res Int. 2017 Mar 16;2017:4826030. doi: 10.1155/2017/4826030 (PMC5376426; doi:10.1155/2017/4826030)
Supplement: Supplementary file 1 — Additional file 1-Table S1: Binding of epitopes of IvAgNP from Influenza virus against MHC II alleles. Additional file 2-Table S2: Binding affinity of predicted strongest epitope 301-IDPFRLLQNSQVYSL-315 from IvAgNP to MHC alleles. Additional file 3-Table S3: Accession numbers of antigenic Ag85B sequences of Mycobacterium pathogenic strains found in protein database. Additional file 4-Figure S1: Multiple alignment of protein sequences of IvAgNP from Influenza virus. Additional file 5-Table S4: Hydrogen Bonds and binding statistics of epitope 181-QQFIYAGSLSALLDP-195 from Ag85B with MHC II complexes. Additional file 6-Figure S2: Docking of predicted strongest epitope 301-IDPFRLLQNSQVYSL-315 from IvAgNP with HLA-DRB1∗0101 (A), DRB1∗0901 (B), DRB1∗0404 (C) and DRB1∗0701 (D). Additional file 7-Table S5: Hydrogen Bonds and binding statistics of predicted strongest epitope 301-IDPFRLLQNSQVYSL-315 from IvAgNP with MHC II complexes. Additional file 8-Table S6: Hydrogen Bonds and binding statistics of epitope 181-QQFIYAGSLSALLDP-195 from Ag85B with MHC I complexes. Additional file 9-Figure S3: Docking of control epitope 380-ELRSRYWAIRTRSG-393 from IvAgNP with HLA-B8 (A) and B27 (B). Additional file 10-Table S7: Hydrogen Bonds and binding statistics of control epitope 380-ELRSRYWAIRTRSG-393 from IvAgNP with MHC I complexes. Additional file 11-Figure S4: Docking of predicted strongest epitope 301-IDPFRLLQNSQVYSL-315 from IvAgNP with HLA-A2 (A), A3 (B), B27 (C), B39 (D) and B58 (E). Additional file 12-Table S8: Hydrogen Bonds and binding statistics of predicted strongest epitope 301-IDPFRLLQNSQVYSL-315 from IvAgNP with MHC I complexes. Additional file 13-Figure S5: Multiple alignments of amino acid sequences of HLA-A and HLA-B series. [file 4826030.f1.pdf]

Additional Files

Additional file 1-Table S1: Binding of epitopes of IvAgNP from Influenza virus against MHC II alleles

Additional file 2-Table S2: Binding affinity of predicted strongest epitope 301-IDPFRLLQNSQVYSL-315 from IvAgNP to MHC alleles

Additional file 3-Table S3: Accession numbers of antigenic Ag85B sequences of *Mycobacterium* pathogenic strains found in protein database

Additional file 4-Figure S1: Multiple alignment of protein sequences of IvAgNP from Influenza virus

Additional file 5-Table S4: Hydrogen Bonds and binding statistics of epitope 181-QQFIYAGSLSALLDP-195 from Ag85B with MHC II complexes

Additional file 6-Figure S2: Docking of predicted strongest epitope 301-IDPFRLLQNSQVYSL-315 from IvAgNP with HLA-DRB1*0101 (A), DRB1*0901 (B), DRB1*0404 (C) and DRB1*0701 (D)

Additional file 7-Table S5: Hydrogen Bonds and binding statistics of predicted strongest epitope 301-IDPFRLLQNSQVYSL-315 from IvAgNP with MHC II complexes

Additional file 8-Table S6: Hydrogen Bonds and binding statistics of epitope 181-QQFIYAGSLSALLDP-195 from Ag85B with MHC I complexes

Additional file 9-Figure S3: Docking of control epitope 380-ELRSRYWAIRTRSG-393 from IvAgNP with HLA-B8 (A) and B27 (B)

Additional file 10-Table S7: Hydrogen Bonds and binding statistics of control epitope 380-ELRSRYWAIRTRSG-393 from IvAgNP with MHC I complexes

Additional file 11-Figure S4: Docking of predicted strongest epitope 301-IDPFRLLQNSQVYSL-315 from IvAgNP with HLA-A2 (A), A3 (B), B27 (C), B39 (D) and B58 (E)

Additional file 12-Table S8: Hydrogen Bonds and binding statistics of predicted strongest epitope 301-IDPFRLLQNSQVYSL-315 from IvAgNP with MHC I complexes

Additional file 13-Figure S5: Multiple alignments of amino acid sequences of HLA-A and HLA-B series

Additional file 1-Table S1: Binding of epitopes of IvAgNP from Influenza virus against MHC II molecules[a]

MHC II molecule	Strongest epitope				No of strong binding epitopes	No of weak binding epitopes
	Start position	Full peptide (15 mer)	Core peptide (9 mer)	Affinity (nM)		
HLA-DRB1*0101	301	IDPFRLLQNSQVYSL	FRLLQNSQV	3.4	98	212
HLA-DRB1*0301	106	RELILYDKEEIRRIW	ILYDKEEIR	7.6	7	59
HLA-DRB1*0401	441	RTEIIRMMESARPEDDP	IRMMESARP	12	13	139
HLA-DRB1*0404	302	FRLLQNSQVYSLIVGID	FRLLQNSQV	7.4	28	139
HLA-DRB1*0405	299	PFRLLQNSQVY	FRLLQNSQV	18.4	13	109
HLA-DRB1*0701	339	EDLRVLSFIKGTKVLTE	LSFIKGTKV	4	39	112
HLA-DRB1*0901	442	IIRMMESARPEDV	IRMMESARP	27.6	10	110
HLA-DRB1*1101	190	VMELVRMIKRGINDRT	LVRMIKRGI	8.6	36	117
HLA-DRB1*1302	360	RGVQIASNENMETMT	VQIASNENM	41.1	2	57
HLA-DRB1*1501	378	LELRSRYWAIRTRS	TLELRSRYW	26.8	8	103
HLA-DRB3*0101	484	EGSYFFGDNAEEYDN	FFGDNAEEY	16.1	3	46
HLA-DRB4*0101	440	MRTEIIRMMESARPE	IRMMESARP	15.6	9	131
HLA-DRB5*0101	225	ILKGKFQTAAQKAMM	GKFQTAAQK	8.8	52	114
HLA-DPA1*0103 -DPB1*0401	250	NAEFEDLTFLARSAL	FEDLTFLAR	7.8	6	52
HLA-DPA1*0103 -DPB1*0201	247	NPGNAEFEDLTFLAR	FEDLTFLAR	13.8	17	83
HLA-DPA1*0201 -DPB1*0101	250	NAEFEDLTFLARSAL	FEDLTFLAR	14.5	19	105
HLA-DPA1*0301 -DPB1*0402	247	NPGNAEFEDLTFLAR	FEDLTFLAR	24.7	12	39
HLA-DQA1*0101-DQB1*0501	334	HSAAFEDLRVLSFIK	FEDLRVLSF	11.8	35	76
HLA-DQA1*0102-DQB1*0602	332	ACHSAAFEDLRVLSF	HSAAFEDLR	14	5	25
HLA-DQA1*0102-DQB1*0602	439	DMRTEIIRMMESARP	TEIIRMMES	11.2	42	138
HLA-DQA1*0501-DQB1*0201	483	NEGSYFFGDNAEEYD	FFGDNAEEY	13	3	23
HLA-DQA1*0501-DQB1*0301	170	STLPRRSGAAGAAVK	SGAAGAAVK	7.8	56	126

[a]NetMHCII 2.2 server was used for predication of epitopes from IvAgNP that showed strong binding affinity with 22 MHC II molecules. Affinity, below 50 nM is considered strong immunogenicity. The strongest epitope bound to HLA-DRB1*0101 MHC II molecule is shaded.

Additional file 2-Table S2: Binding affinity of predicted strongest epitope 301-IDPFRLLQNSQVYSL-315 from IvAgNP to MHC alleles [a]

	Promiscuous peptide sequence		
MHC II Alleles	Full peptide (15 mer)	Core peptide (9 mer)	Affinity (nM)
HLA-DRB1*0101	IDPFRLLQNSQVYSL	FRLLQNSQV	3.4
HLA-DRB1*0404	IDPFRLLQNSQVYSL	FRLLQNSQV	7.8
HLA-DRB1*0405	IDPFRLLQNSQVYSL	FRLLQNSQV	21.1
HLA-DRB1*0701	IDPFRLLQNSQVYSL	FRLLQNSQV	32.6
HLA-DRB1*0901	IDPFRLLQNSQVYSL	FRLLQNSQV	39.4

[a]MHC allele was predicted by NetMHCII 2.2 servers. Binding affinity below 50 nM is considered high immunogenicity.

Additional file 3-Table S3: Accession numbers of all antigenic Ag85-B sequences of *Mycobacterium* pathogenic strains found in protein database

Mycobacterium pathogenic strain	Accession number	Number of identical sequences	Number of amino acid	Accession number of Identical sequences
M. Tuberculosis_2 CDC1551	NP_336393.1	2	374	NP_336393.1, AAK46207.1
M. Bovis_1 BCG str. Pasteur 1173P2	YP_978013.1	10	325	YP_978013.1, YP_005171366.1, AET19183.1, CCC64491.1, ADD50055.1, YP_002644961.1, BAH26193.1, A1KJU9.1, CAL71910.1, CAA44268.1
M. Tuberculosis_4 SUMu003	ZP_07422952.2	12	322	ZP_07422952.2, YP_005913165.1, YP_005909553.1, BAL65871.1, AEJ50558.1, AEJ46932.1, EGB28751.1, ZP_074402622, ZP_07436007.2, EFP38634.1, EFP30851.1, EFP19521.1
M. Tuberculosis_5 H37Ra	ZP_02552646.1	25	310	ZP_02552646.1, GAA45592.1, ZP_07815649.1, EFP54785.1, EFP51110.1, EFP47207.1, EFP43274.1, EFP34332.1, EFP27102.1, EFP23309.1, EFP16040.1, EFO74877.1, ZP_07493616.1, ZP_07489096.1, ZP_07484878.1, ZP_07480643.1, ZP_07444836.1, ZP_07431632.1, ZP_07427317.1, ZP_07418226.1, ZP_07414444.1, ZP_06960553.1, ZP_06952228.1, ZP_06802485.1, ZP_05141345.1
M. bovis_2	AAA25359.1	9	323	AAA25359.1, AAQ77588.1, AAN20429.1, AAN19158.1, AAE80437.1, AAE55762.1, AAE40111.1, AAE36056.1, AAE31577.1
M. Tuberculosis_3	1F0P|A	1	285	1F0P|A
M. Intracellulare_3	BAA03243.1	1	264	BAA03243.1
M. Intracellulare_1 ATCC 13950	YP_005338175.1	6	315	YP_005338175.1, YP_005348339.1, YP_005343334.1, AFC54018.1, AFC49013.1, AFC43854.1
M. Tuberculosis_6	1F0N_A	1	285	1F0N_A
M. kansasii ATCC 12478	ZP_04749192.1	5	325	ZP_04749192.1, AAR68430.1, AAE27820.1, P21160.1 CAA37868.1
M. avium_1	Q06947.1	3	330	AAM58787.1, Q06947.1, CAA45032.1
M. Tuberculosis_7	AAO62005.1	1	325	AAO62005.1
M. intracellulare_2 ATCC 13950	BAA03981.1	1	330	BAA03981.1
M. avium_2 subsp. Paratuberculosis	AAM21939.1	1	330	AAM21939.1
M. Tuberculosis_1 H37Rv	NP_216402.1	72	325	P0C5B9.1, YP_006515286.1, YP_005916959.1, YP_004745346.1, YP_004723581.1, YP_006473577.1, YP_005308170.1, YP_005923115.1, YP_005100587.1, ZP_07012790.1, ZP_06521415.1, ZP_06517371.1, ZP_06509845.1, ZP_06454795.1, ZP_06450191.1, ZP_06513358.1, ZP_06505060.1, ZP_06437244.1, ZP_06433108.1, ZP_04980753.1, ZP_04925390.1, YP_003032071.1, YP_001283215.1, YP_001287851.1, A5U3Q3.1, P0C2T2.1, NP_855570.1, AFE13119.1, CCE37368.1, AEN00338.1, EGE50422.1, EFD43577.1, EFD17659.1, EFD13523.1, EFD73559.1, EFD47366.1, AEB04248.1, EFI30469.1, EFD77569.1, EFD61996.1, EFD53698.1, EFD58483.1, ACT25176.1, ABQ73653.1, EBA42266.1, AFN49815.1, ABR06249.1, EAY60132.1, AFM49500.1, CCC26978.1, CCC44233.1, ADS25363.1, AAC44294.1, ADD50054.1, ADF19013.1, ABY17327.1, ABY08008.1, CAD94621.1, CAB10044.1, ACC93938.1, CAA44269.1, ABE22073.1, AAQ77586.1, AAN19156.1, AAN20427.1, AAM58786.1, AAE80435.1, AAE55760.1, AAE40109.1, AAE36054.1, AAE31575.1

Additional file 4-Figure S1: Multiple alignment of protein sequences of IvAgNP from Influenza virus[a]

```
Influenza A virus_1    298 LVGIDPFRLLQNSQVYSLIRPNENPAHKSQLVWMACHSAAFEDLRVLSFIKGTKVLPRGKLSTRGVQIASNENMETMESSTLELRSRYWAIRTRSGGNTNQ  398
Influenza A virus_2    298 LVGIDPFKLLQNSQVYSLIRPNENPAHKSQLVWMACNSAAFEDLRVLSFIRGTKVSPRGKLSTRGVQIASNENMDAMESSTLELRSRYWAIRTRSGGNTNQ  398
Influenza A virus_3    298 LVGIDPFRLLQNSQVFSLIRPNENPAHKSQLVWMACHSAAFEDLRVSSFIRGTRVLPRGQLSTRGVQIASNENMETMNSSTLELRSKYWAIRTRSGGNTNQ  398
Influenza A virus_4    298 LVGIDPFRLLQNSQVFSLIRPNENPAHKSQLVWMACHSAAFEDLRVSSFIRGTRVVPRGQLSTRGVQIASNENMETMDSSTLELRSRYWAIRTKSGGNTNQ  398
Influenza A virus_5    298 LVGIDPFRLLQNSQVFSLIRPNENSAHKSQLVWMACHSAAFEDLRVSSFIRGTKVVPRGKLSTRGVQIASNENMETMDSTTLELRSRYWAIRTRSGGNTNQ  398
Influenza A virus_6    298 LVGIDPFRLLQNSQVFSLIRPNENPAHKSQLVWMACHSAAFEDLRVSSFIRGTRVVPRGQLSTRGVQIASNENMDAIDSSTLELRSRYWAIRTRSGGNTNQ  398
Influenza A virus_7    298 LVGIDPFKLLQTSQVYSLIRPNENPAHKSQLVWMACNSAAFEDLRVSSFIRGTKVIPRGKLSTRGVQIASNENMDTMGSSTLELRSRYWAIRTRSGGNTNQ  398
Influenza A virus_8    298 LVGIDPFKLLQTSQVYSLIRPNENPAHKSQLVWMACNSAAFEDLRVSSFIRGTKVIPRGKLSTRGVQIASNENMDTMGSSTLELRSRYWAIRTRSGGNTNQ  398
Influenza A virus_9    298 LVGIDPFRLLQNSQVFSLIRPNENPAHKSQLVWMACHSAAFEDLRVSSFIRGTRVLPRGQLSTRGVQIASNENMETMNSSTLELRSRYWAIRTRSGGNTNQ  398
Influenza A virus_10   298 LVGIDPFRLLQNSQVFSLIRPNENPAHKSQLVWMACHSAAFEDLRVSSFIRGTRVLPRGQLSTRGVQIASNENMETMNSSTLELRSKYWAIRTRSGGNTNQ  398
Influenza A virus_11   298 LVGIDPFRLLQNSQVFSLIRPNENPAHKSQLVWMACHSAAFEDLRVSNFIRGTRVVPRGKLSTRGVQIASNENMETMDSSTLELRSRYWAIRTRSGGNTNQ  398
Influenza A virus_12   298 LVGIDPFKLLQNSQVYSLIRPNENPAHKSQLVWMACNSAAFEDLRLLSFIRGTKVSPRGKLSTRGVQIASNENMDTMESSTLELRSRYWAIRTRSGGNTNQ  398
Influenza A virus_13   298 LVGIDPFKLLQNSQVYSLIRPNENPAHKSQLVWMACNSAAFEDLRLLSFIRGTKVSPRGKLSTRGVQIASNENMDTMESSTLELRSRYWAIRTRSGGNTNQ  398
Influenza A virus_14   298 LVGIDPFKLLQNSQVYSLIRPNENPAHKSQLVWMACHSAAFEDLRLLSFIRGTKVSPRGKLSTRGVQIASNENMDTMESSTLELRSRYWAIRTRSGGNTNQ  398
Influenza A virus_15   298 LVGIDPFKLLQNSQVYSLIRPNENPAHKSQLVWMACHSAAFEDLRLLSFIRGTKVSPRGKLSTRGVQIASNENMDTMESSTLELRSRYWAIRTRSGGNTNQ  398
Influenza A virus_16   298 LVGIDPFKLLQNSQVYSLIRPNENPAHKSQLVWMACHSAAFEDLRLLSFIRGTKVSPRGKLSTRGVQIASNENMDTMESSTLELRSRYWAIRTRSGGNTNQ  398
Influenza A virus_17   298 LVGIDPFKLLQNSQVYSLIRPNENPAHKSQLVWMACHSAAFEDLRLLSFIRGTKVSPRGKLSTRGVQIASNENMDNMESSTLELRSRYWAIRTRSGGNTNQ  398
Influenza A virus_18   298 LVGIDPFRLLQNSQVFSLIRPNENPAHKSQLVWMACHSAAFEDLRVSSFIRGTRVVPRGQLSTRGVQIASNENMETMDSSTLELRSRYWAIRTRSGGNTNQ  398
Influenza A virus_19   298 LVGIDPFRLLQNSQVFSLIRPNENPAHKSQLVWMACHSAAFEDLRVSSFIRGTRVVPRGQLSTRGVQIASNENMETMDSRTLELRSRYWAIRTRSGGNTNQ  398
Influenza A virus_20   298 LVGIDPFKLLQNSQVYSLIRPNENPAHKSQLVWMACHSAAFEDLRLLSFIRGTKVSPRGKLSTRGVQIASNENMDTMESSTLELRSRYWAIRTRSGGNTNQ  398
Influenza A virus_21   298 LVGIDPFRLLQNSQVFSLIRPNENPAHKSQLVWMACHSAAFEDLRVSSFIRGTRVVPRGQLSTRGVQIASNENMETMDSSTLELRSRYWAIRTRSGGNTNQ  398
Influenza A virus_22   298 LVGIDPFRLLQNSQVFSLIRPNENPAHKSQLVWMACHSAAFEDLRVSSFIRGTRVAPRGQLSTRGVQIASNENMETMDSSTLELRSRYWAIRTRSGGNTNQ  398
Influenza A virus_23   298 LVGIDPFKLLQNSQVYSLIRPNENPAHKSQLVWMACHSAAFEDLRLLSFIRGTKVSPRGKLSTRGVQIASNENMDTMESSTLELRSRYWAIRTRSGGNTNQ  398
Influenza A virus_24   298 LVGIDPFRLLQNSQVFSLIRPNENPAHKSQLVWMACHSPAFEDLRVSSFIRGTRVVPRGQLSTRGVQIASNENMETMDSSTLELRSRYWAIRTRSGGNTNQ  398
Influenza A virus_25   298 LVGIDPFRLLQNSQVFSLIRPNENPAHKSQLVWMACHSAAFEDLRVSSFIRGTRVVPRGQLSTRGVQIASNENMETMDSSTLELRSRYWAIRTRSGGNTNQ  398
Influenza A virus_26   298 LVGIDPFKLLQNSQVFSLIRPNENPAHKSQLVWMACHSAAFEDLRVSGFIRGKKVVPRGKLSTRGVQIASNENVEAMDSSTLELRSRYWAIRTRSGGNTNQ  398
Influenza A virus_27   298 LVGIDPFRLLQNSQVFSLIRPNENPAHKSQLVWMACHSAAFEDLRVSSFIRGTRLVPRGQLSTRGVQIASNENMEAMDSNTLELRSRYWAIRTRSGGNTNQ  398
Influenza A virus_28   298 LVGIDPFRLLQNSQVFSLIRPNENPAHKSQLVWMACHSAAFEDLRVSSFIRGTRVLPRGQLSTRGVQIASNENMETMNSSTLELRSKYWAIRTRSGGNTNQ  398
Influenza A virus_29   298 LVGIDPFRLLQNSQVFSLIRPNENPAHKSQLVWMACHSAAFEDLRVSSFIRGTRVLPRGQLSTRGVQIASNENMETMNSSTLELRSKYWAIRTRSGGNTNQ  398
Influenza A virus_30   298 LVGIDPFRLLQNSQVYSLIRPNENPAHKSQLVWMACHSAAFEDLRVLSFIKGTKVLPRGKLSTRGVQIASNENMETMESSTLELRSRYWAIRTRSGGNTNQ  398
Influenza A virus_31   298 LVGIDPFRLLQNSQVFSLIRPNENPAHKSQLVWMACHSAAFEDLRVSSFIRGKRVVPRGQLSTRGVQIASNENMETMDSSTLELRSRYWAIRTRSGGNTNQ  398
Influenza A virus_32   298 LVGIDPFKLLQNSQVYSLIRPNENPAHKSQLVWMACNSAAFEDLRVSSFIRGTKVIPRGKLSTRGVQIASNENMDTMGSSTLELRSRYWAIRTRSGGNTNQ  398
Influenza A virus_33   298 LVGIDPFRLLQNSQVFSLIRPNENPAHKSQLVWMACHSAAFEDLRVSSFIRGTRVVPRGQLSTRGVQIASNENMETMDSSTLELRSRYWAIRTRSGGNTNQ  398
Influenza A virus_34   298 LVGIDPFRLLQNSQVFSLIRPKENPAHKSQLVWMACHSAAFEDLRVSSFIRGTRVVPRGQLSTRGFQIASNENMEAMDSSTLELRSRYWAIRTRSGGNTNQ  398
Influenza A virus_35   298 LVGIDPFRLLQNSQVFSLIRPNENPAHKSQLVWMACHSAAFEDLRVSSFIRGTRVVPRGQLSTRGVQIASNENMETMDSSTLELRSRYWAIRTRSGGNTNQ  398
Influenza A virus_36   298 LVGIDPFKLLQNSQVYSLIRPNENPAHKSQLVWMACNSAAFEDLRLLSFIRGTKVFPRGKLSTRGVQIASNENMDTMESSTLELRSRYWAIRTRSGGNTNQ  398
Influenza A virus_37   298 LVGIDPFKLLQNSQVYSLIRPNENPAHKSQLVWMACNSAAFEDLRLLSFIRGTKVSPRGKLSTRGVQIASNENMDTMESSTLELRSRYWAIRTRSGGNTNQ  398
Influenza A virus_38   298 LVGIDPFKLLQNSQIFSLIRPKENPAHKSQLVWMACHSAAFEDLRVSNFIRGTKVIPRGQLATRGVQIASNENMETIDSSTLELRSRYWAIRTRSGGNTSQ  398
Influenza A virus_39   298 LVGIDPFRLLQNSQVFSLIRPNENPAHKSQLVWMACHSAAFEDLRVSSFIRGTRVIPRGQLSTRGVQIASNENMETMDSSTLELRSRYWAIRTRSGGNTNQ  398
Influenza A virus_40   298 LVGIDPFKLLQNSQVYSLIRPNENPAHKSQLVWMACHSAAFEDLRLLSFIRGTKVCPRGKLSTRGVQIASNENMDTMESSTLELRSRYWAIRTRSGGNTNQ  398
Influenza A virus_41   298 LVGIDPFKLLQNSQVYSLIRPNENPAHKSQLVWMACHSAAFEDLRLLSFIRGTKVSPRGKLSTRGVQIASNENMDTMESSTLELRSRYWAIRTRSGGNTNQ  398
Influenza A virus_42   298 LVGIDPFRLLQNSQVFSLIRPNENPAHKSQLVWMACHSAAFEDLRVSSFIRGTRVLPRGQLSTRGVQIASNENMETMNSSTLELRSKYWAIRTRSGGNTNQ  398
Influenza A virus_43   298 LVGIDPFKLLQNSQVYSLIRPNENPAHKSQLVWMACHSAAFEDLRLFSFIRGTKVCPRGKLSTRGVQIASNENMDTMESSTLELRSRYWAIRTRSGGNTNQ  398
Influenza A virus_44   298 LVGIDPFKLLQNSQVYSLIRPNENPAHKSQLVWMACHSAAFEDLRLLSFIRGTKVCPRGKLSTRGVQIASNENMDNMESSTLELRSRYWAIRTRSGGNTNQ  398
Influenza A virus_45   298 LVGIDPFRLLQNSQVFSLIRPNENPAHKSQLVWMACHSAAFEDLRVSSFIRGARVLPRGQLSTRGVQIASNENMETMSSSTLELRSKYWAIRTRSGGNTNQ  398
Influenza A virus_46   298 LVGIDPFKLLQNSQVYSLIRPNENPAHKSQLVWMACHSAAFEDLRLLSFIRGTKVSPRGKLSTRGVQIASNENMDNMESSTLELRSRYWAIRTRSGGNTNQ  398
Influenza A virus_47   298 LVGIDPFRLLQNSQVFSLIRPNENPAHKSQLVWMACHSAAFEDLRVSSFIRGTKVVPRGQLSTRGVQIASNENMETMDSSTLELRSKYWAIRTRSGGNTNQ  398
Influenza A virus_48   298 LVGIDPFRLLQNSQVFSLIRPNENPAHKSQLVWMACHSAAFEDLRVSSFIRGTRVVPRGQLSTRGVQIASNENMETMDSSTLELRSRYWAIRTRSGGNTNQ  398
Influenza A virus_49   298 LVGIDPFKLLQNSQVYSLIRPNENPAHKSQLVWMACHSAAFEDLRLLSFIRGTKVSPRGKLSTRGVQIASNENMDNMESSTLELRSRYWAIRTRSGGNTNQ  398
Influenza A virus_50   298 LVGIDPFKLLQNSQIFSLIRPKENPAHKSQLVWMACHSAAFEDLRVLNFIRGTKVIPRGQLATRGVQIASNENMETIDSSTLELRSRYWAIRTRSGGNTSQ  398
Influenza A virus_51   298 LVGIDPFKLLQNSQVYSLIRPNENPAHKSQLVWMACHSAAFEDLRLLSFIRGTKVSPRGKLSTRGVQIASNENMDTMESSTLELRSRYWAIRTRSGGNTNQ  398
Influenza A virus_52   298 LVGIDPFKLLQNSQVYSLIRPNENPAHKSQLVWMACHSAAFEDLRLLSFIRGTKVSPRGKLSTRGVQIASNENMDNMESSTLELRSRYWAIRTRSGGNTNQ  398
Influenza A virus_53   298 LVGIDPFKLLQNSQVYSLIRPNENPAHKSQLVWMACHSAAFEDLRLLSFIRGTKVSPRGKLSTRGVQIASNENMDNMESSTLELRSRYWAIRTRSGGNTNQ  398
Influenza A virus_54   298 LVGIDPFRLLQNSQVFSLIRPNENPAHKSQLVWMACHSAAFEDLRVSSFIRGTRVVPRGQLSTRGVQIASNENMETMDSNTLELRSRYWAIRTRSGGNTSQ  398
Influenza A virus_55   298 LVGIDPFRLLQNSQVFSLIRPNENPAHKSQLVWMACHSAAFEDLRVSSFIRGTRVVPRGQLSTRGVQIASNENMETMDSSTLELRSRYWAIRTRSGGNTNQ  398
Influenza A virus_56   298 LVGIDPFRLLQNSQVFSLIRPNENPAHKSQLVWMACHSAAFEDLRVSSFIRGTRVIPRGQLSTRGVQIASNENMETMDSSTLELRSRYWAIRTRSGGNTNQ  398
Influenza A virus_57   298 LVGIDPFRLLQNSQVYSLIRPNENPAHKSQLVWMGCHSAAFEDLRVLSFIKGTKVVPRGKLSTRGVQIASNENMETMESSTLELRSRYWAIRTRSGGNTNQ  398
Influenza A virus_58   130 LVGIDPFRLLQNSQVFSLIRPNENPAHKSQLVWMACHSAAFEDLRVSSFIRGTRVVPRGQLSTRGVQIASNENMEAMDSNTLELRSRYWAIRTRSGGNTNQ  230
Influenza A virus_59   298 LVGIDPFRLLQNSQVFSLIRPNENPAHKSQLVWMACHSAAFEDLRVSSFIRGTRVIPRGQLSTRGVQIASNENVEAMDSSTLELRSRYWAIRTRSGGNTNQ  398
Influenza A virus_60   298 LVGIDSFRLLQNSQVFSLIRPNENPAHKSQLVWMACHSAAFEDLRVSSFIRGTRVVPRGQLSTRGVQIASNENMETMDSSTLELRSRYWAIRTRSGGNTNQ  398
Influenza A virus_61   298 LVGIDPFRLLQNSQVFSLIRPNENPAHKSQLVWMACHSAAFEDLRVSSFIRGTRVVPRGQLSTRGVQIASNENMETMDSSTLELRSRYWAIRTRSGGNTNH  398
Influenza A virus_62   298 LVGIDPFRLLQNSQVFSLIRPNENPAHKSQLVWMACHSAAFEDLRVSSFIRGTRVVPRGQLSTRGVQIASNENMETMDSSTLELRSRYWAIRTRSGGNTNQ  398
Influenza A virus_63   298 LVGIDPFRLFQNSQVFSLIRPNENPAHKSQLVWMACHSAAFEDLRVSSFIRGTRVVPRGQLSTRGVQIASNENMETMDSSTLELRSRYWAIRTRSGGNTNQ  398
Influenza A virus_64   298 LVGIDPFRLLQNSQVFSLIRPNENPAHKSQLVWMACHSAAFEDLRVSSFIRGTRVVPRGQLSTRGVQIASNENMETMDSSTLELRSRYWAIRTRSGGNTNQ  398
Influenza A virus_65   298 LVGIDPFRLLQNSQVFSLIRPNENPAHKSQLVWMACHSAAFEDLRVSSFIRGTRVVPRGQLSTRGIQIASNENMETMDSSTLELRSRYWAIRTRSGGNTNQ  398
Influenza A virus_66   298 LVGIDPFRLLQNSQVFSLIRPNENPAHKSQLVWMACHSAAFEDLRVSSFIRGTRVVPRGQLSTRGIQIASNENMETMDSSTLELRSRYWAIRTRSGGNTNQ  398
Influenza A virus_67   298 LVGIDPFRLLQNSQVYSLIRPNENPAHKSQLVWMACHSAAFEDLRVLSFIKGTKVLPRGKLSTRGVQIASNENMETMESSTLELRSRYWAIRTRSGGNTNQ  398
Influenza A virus_68   296 LVGIDPFRLLQNSQVFSLIRPNENPAHKSQLVWMACHSAAFEDLRVSSFIRGTRVVPRGQLSTRGVQIASNENMEVMDSNTLELRSRYWAIRTRSGGNTNQ  396
Influenza A virus_69   298 LVGIDPFRLLQNSQVFSLIRSNENPAHKSQLVWMACHSAAFEDLRVSSFIRGTRVIPRGQLSTRGVQIASNENMETIDSSTLELRSRYWAIRTRSGGNTNQ  398
Influenza A virus_70   298 LVGIDPFRLLQNSQVFSLIRPNENPAHKSQLVWMACHSAAFEDLRVSSFIRGTRVVPRGQLSTRGVQIASNENMEAMDSNTLELRSRYWAIRTRSGGNTSQ  398
Influenza A virus_71   298 LVGIDPFRLLQNSQVFSLIRPNENPAHKSQLVWMACHSAAFEDLRVSSFIRGTRVVPRGQLSTRGVQIASNENMEAMDSNTLELRSRYWAIRTRSGGNTNQ  398
Influenza A virus_72   298 LVGIDPFRLLQNSQVFSLIRPNENPAHKSQLVWMACHSAAFEDLRVSSFIRGTRVVPRGQLSTRGVQIASNENMEAMDSNTLELRSRYWAIRTRSGGNTSQ  398
Influenza A virus_73   298 LVGIDPFRLLQNSQVYSLIRPNENPAHKSQLVWMACHSAAFEDLRVLSFIKGTKVLPRGKLSTRGVQIASNENMETMESSTLELRSRYWAIRTRSGGNTNQ  398

                           *****.*:*:*.**::*:**.:**.*********.*:*.******: .**:* :: ***:*:***.*******.....*.******.******.*****..
```

[a]The control epitope 380-ELRSRYWAIRTRSG-393 and the predicted strongest epitope 301-IDPFRLLQNSQVYSL-315 is shown in the box. The epitope sequences from Influenza A virus is underlined. Non-identical epitope sequences are shaded and the amino acid residues for which the epitope varies are bold, italic and underlined

Additional file 5-Table S4: Hydrogen Bonds and binding statistics of epitope 181-QQFIYAGSLSALLDP-195 with MHC II complexes[a]

Epitope-MHC II complex	No. of H-bonds	Binding energy (kcal/mol)	Receptor residues in MHC I	Ligand residues in epitope	Interacting atoms	H-bond distance (Å)
Epitope-HLA-DRB1*0101	9	-181.93	W61	Q1	H-O	2.55
			Q64	Q1	H-O	1.85
			Q70	Y5	H-O	2.43
			R71	Q2	H-O	1.84
			R71	Q2	H-O	1.97
			Y78	L9	H-O	1.65
			N82	S10	H-O	2.11
			Q64	Q1	O-H	2.25
			T77	Y5	O-H	2.56
Epitope-HLA-DRA	6	-204.17	N69	L13	H-O	2.41
			N69	P15	H-O	2.45
			N69	L13	H-O	2.18
			E11	S10	O-H	2.38
			F48	Q2	O-H	2.48
			F48	Q2	O-H	2.64
Epitope-HLA-DRB1*0404	9	-162.42	H13	Q1	H-O	2.64
			Y60	L13	H-O	2.76
			D28	Q1	O-H	2.38
			D28	Q1	O-H	2.65
			Q64	D14	O-H	2.78
			D66	S10	O-H	2.38
			D66	S8	O-H	2.36
			Q70	Y5	O-H	2.30
			T77	Q2	O-H	2.14
Epitope-HLA-DRB1*0701	8	-179.42	C15	P15	H-O	2.14
			S60	Q1	H-O	2.62
			W61	I4	H-O	2.46
			R71	L9	H-O	2.38
			Q74	D14	H-O	1.92
			N82	D14	H-O	2.19
			N82	L13	H-O	2.14
			Q64	Q1	O-H	2.76
Epitope-HLA-DRB1*0901	6	-178.35	Y26	D14	H-O	2.22
			Y26	D14	H-O	2.70
			R71	L13	H-O	2.32
			R71	L13	H-O	2.53
			T77	P15	H-O	2.59
			Y32	I4	O-H	2.40
Epitope-HLA-DPB1*0101	5	-159.39	N62	S8	H-O	2.66
			N62	S8	H-O	2.01
			Y9	S10	O-H	2.50
			G58	S8	O-H	2.28
			A64	Q2	O-H	2.55
Epitope-HLA-DQB1*0301	9	-202.60	Y37	L13	H-O	2.38
			Y37	D14	H-O	2.34
			W61	A11	H-O	2.79
			Q64	S10	H-O	2.47
			R70	L9	H-O	2.52
			N82	Q1	O-H	2.70
			Q64	S10	O-H	2.13
			T77	Q1	O-H	2.20
			N82	Q1	O-H	2.10

[a]Hydrogen bonding distance and binding energy were analyzed in Molsoft ICM-pro 3.5 computer program. Fifteen amino acid residues in the epitope (181-QQFIYAGSLSALLDP-195) have been numbered 1-15.

Additional file 6-Figure S2: Docking of predicted strongest epitope 301-IDPFRLLQNSQVYSL-315 from IvAgNP with HLA-DRB1*0101 (A), DRB1*0901 (B), DRB1*0404 (C) and DRB1*0701 (D)[a]

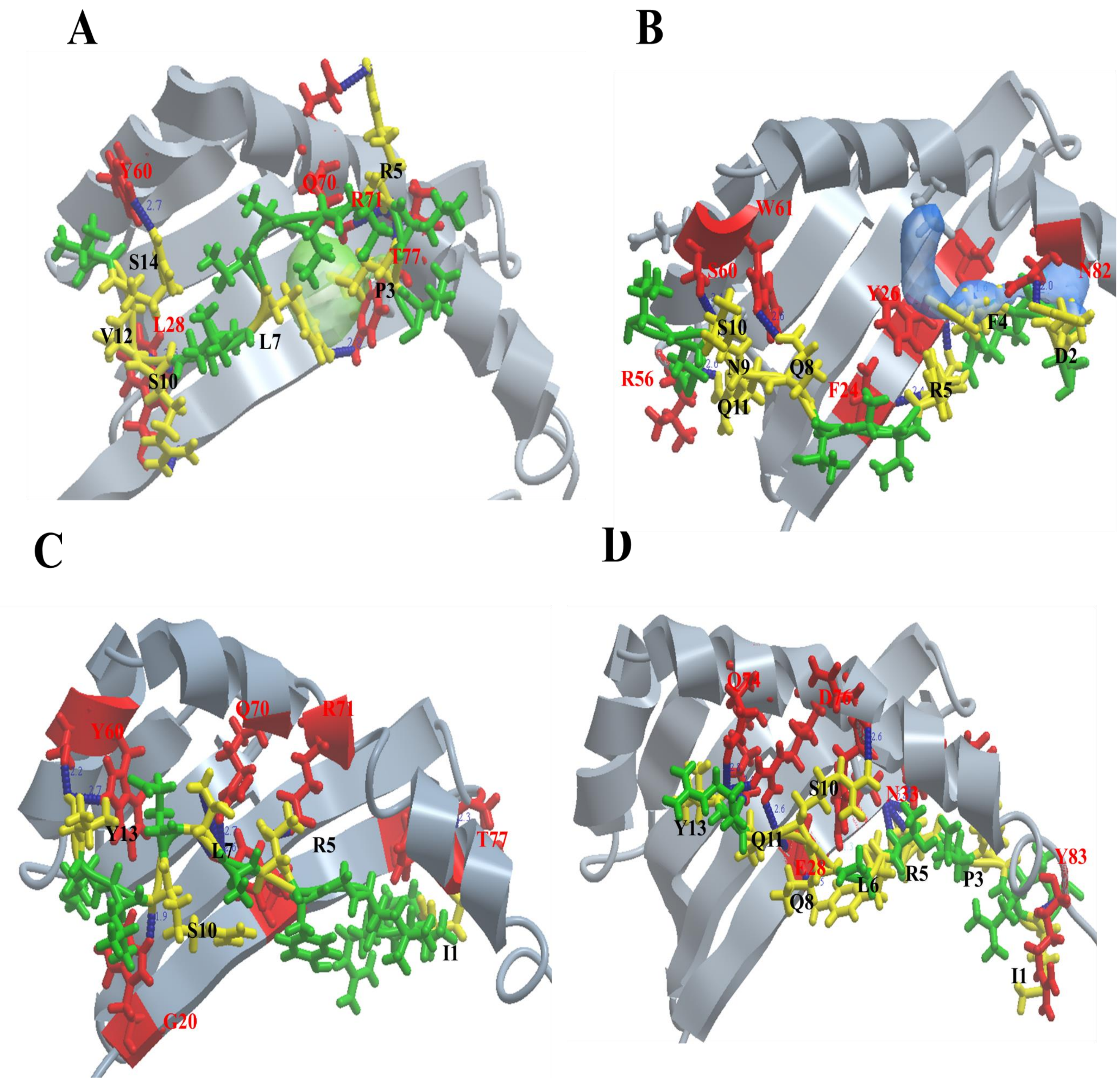

[a]MHC II structures are shown in grey ribbon and the amino acid residues involved in the H-bonding interaction are shown as red sticks and labeled red. The epitope is shown as in green ribbon and the amino acid residues involved in the H-bonding network are shown as yellow sticks and labeled black. MHC II binding pocket(s) are shown as electrostatic sphere. H-bonds are displayed as blue spheres and the H-bonding distances are labeled blue. H-bonding interaction between the amino acid residues of the epitope and those of the MHC II are detailed in additional file 7- Table S5.

Additional file 7-Table S5: Hydrogen Bonds and binding statistics of predicted strongest epitope 301-IDPFRLLQNSQVYSL-315 from IvAgNP with MHC II complexes[a]

Epitope-MHC II complex	No. of H-bonds	Binding energy (kcal/mol)	Receptor residues in MHC I	Ligand residues in epitope	Interacting atoms	H-bond distance (Å)
Epitope-HLA-DRB1*0101	9	-194.87	T77	P3	H-O	2.15
			R71	R5	O-H	2.85
			Q70	R5	H-O	1.23
			R71	P3	H-O	1.54
			R71	L7	O-H	1.91
			Y60	S14	H-O	2.55
			L28	S10	O-H	2.41
			L28	S10	O-H	2.05
			L28	V12	O-H	2.16
Epitope-HLA-DRB1*0901	9	-211.17	N82	D2	H-O	1.41
			N82	F4	O-H	1.45
			H28	F4	H-O	2.18
			Y26	R5	O-H	2.48
			F24	R5	H-O	2.88
			W61	Q8	O-H	2.64
			S60	S10	H-O	2.14
			R56	N9	H-O	2.13
			R56	Q11	H-O	2.12
Epitope-HLA-DRB1*0404	7	-189.62	T77	I1	H-O	2.04
			R71	R5	H-O	2.16
			Q70	L7	O-H	2.28
			G20	S10	O-H	1.65
			Y60	Y13	H-O	1.88
			D28	R5	O-H	2.08
			D28	R5	O-H	2.06
Epitope-HLA-DRB1*0701	8	-185.42	Y83	I1	H-O	2.04
			N33	P3	O-H	2.12
			N33	R5	H-O	2.36
			D76	S10	O-H	2.08
			Q74	Y13	H-O	1.95
			Q74	Y13	H-O	2.26
			D76	Q11	O-H	2.35
			N33	L6	O-H	2.14

[a]Hydrogen bonding distance and binding energy were analyzed in Molsoft ICM-pro 3.5 computer program. Fifteen amino acid residues in the epitope (301-IDPFRLLQNSQVYSL-315) have been numbered 1-15.

Additional file 8-Table S6: Hydrogen Bonds and binding statistics of epitope 181-QQFIYAGSLSALLDP-195 from Ag85B with MHC I complexes [a]

Epitope-MHC I complex	No. of H-bonds	Binding energy (kcal/mol)	Receptor residues in MHC I	Ligand residues in epitope	Interacting atoms	H-bond distance (Å)
Epitope (183-FIYAGSLSA-191)-HLA-A2	8	-162.01	H70	Y3	H-O	2.79
			Y116	S8	H-O	2.66
			W147	A9	H-O	2.58
			Q155	S6	H-O	1.84
			Y159	F1	H-O	1.92
			E63	F1	O-H	2.70
			Y99	Y3	O-H	2.11
			Q155	S6	O-H	2.22
Epitope (184-IYAGSLSAL-192)-HLA-A24	7	-185.45	K66	Y2	H-O	2.18
			N77	S7	H-O	1.75
			Y84	L9	H-O	2.71
			T143	L9	H-O	2.55
			W147	A8	H-O	1.49
			E63	I1	O-H	2.70
			N77	L9	O-H	1.80
Epitope (181-QQFIYAGSL-189)-HLA-B27	11	-193.62	Y59	L9	H-O	2.61
			R62	G7	H-O	2.07
			R62	S8	H-O	1.95
			Y99	Q1	H-O	1.83
			T143	Q2	H-O	2.01
			W147	S8	H-O	2.50
			Y159	L9	H-O	2.35
			Y171	Q1	H-O	2.14
			E63	L9	O-H	2.05
			D77	Q2	O-H	1.88
			D116	Q1	O-H	2.41
Epitope (181-QQFIYAGSL-189)-HLA-B44	11	-200.13	Y9	Q2	H-O	1.81
			R62	Q1	H-O	1.73
			Y74	Y5	H-O	2.31
			Y99	Q2	H-O	2.39
			T143	S8	H-O	1.72
			Y9	Q2	O-H	1.73
			E63	Q1	O-H	1.90
			E63	Q2	O-H	1.89
			N77	L9	O-H	2.52
			D116	Y5	O-H	2.66
			S167	Q1	O-H	2.67
Epitope (184-IYAGSLSAL-192)-HLA-B39	7	175.42	Y84	L9	H-O	2.28
			K146	L9	H-O	2.44
			W147	S7	H-O	2.79
			W147	A8	H-O	1.82
			S77	L9	O-H	1.97
			Y99	Y2	O-H	2.24
			E152	S5	O-H	2.31

[a]Hydrogen bonding distance and binding energy were analyzed in Molsoft ICM-pro 3.5 computer program. Nine amino acid residues in each 9-mer of epitope have been numbered 1-9.

Additional file 9-Figure S3: Docking of control epitope 380-ELRSRYWAIRTRSG-393 from IvAgNP with HLA-B8 (A) and B27 (B)[a]

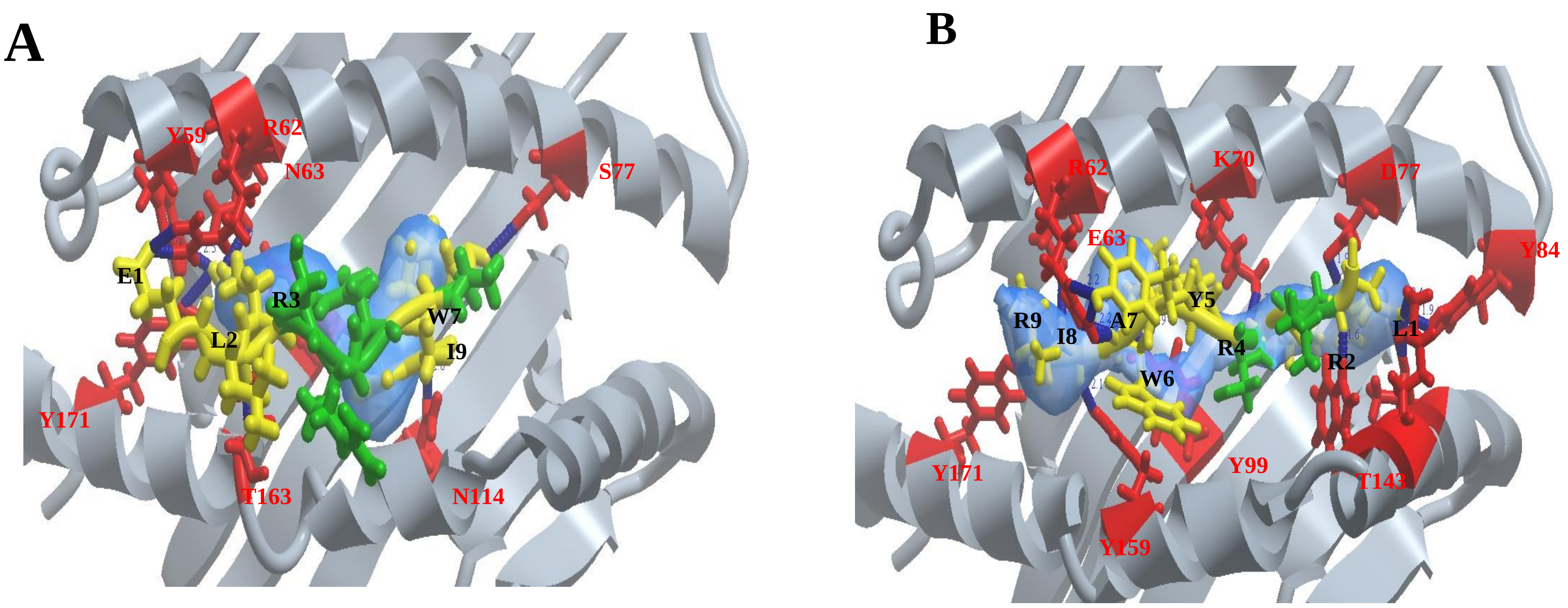

[a]MHC I structures are shown in grey ribbon and the amino acid residues involved in the H-bonding interaction are shown as red sticks and labeled red. The epitope is shown as in green ribbon and the amino acid residues involved in the H-bonding network are shown as yellow sticks and labeled black. MHC I binding pocket(s) are shown as electrostatic sphere. H-bonds are displayed as blue spheres and the H-bonding distances are labeled blue. H-bonding interaction between the amino acid residues of the epitope and those of the MHC I are detailed in additional file 10- Table S7

Additional file 10-Table S7: Hydrogen Bonds and binding statistics of control epitope 380-ELRSRYWAIRTRSG-393 from IvAgNP with MHC I complexes

Epitope-MHC I complex	No. of H-bonds	Binding energy (kcal/mol)	Receptor residues in MHC I	Ligand residues in epitope	Interacting atoms	H-bond distance (Å)
Epitope (380-ELRSRYWAI -388)-HLA-B8	10	-177.86	R62	E1	H-O	2.42
			R62	E1	H-O	1.74
			S77	I9	H-O	2.57
			T163	L2	H-O	2.13
			Y7	R3	O-H	2.45
			Y7	Y6	O-H	1.88
			Y59	R3	O-H	2.29
			N63	R3	O-H	2.22
			N114	W7	O-H	2.67
			Y171	R3	O-H	2.78
Epitope (381-LRSRYWAIR -389)-HLA-B27	14	-234.19	R62	Y5	H-O	2.25
			R62	W6	H-O	2.15
			R62	W6	H-O	2.36
			R62	Y5	H-O	1.82
			K70	R2	H-O	2.65
			Y84	L1	H-O	1.95
			T143	L1	H-O	2.38
			W147	L1	H-O	1.56
			Y159	A7	H-O	2.07
			Y171	R9	H-O	1.89
			E63	R9	O-H	2.75
			D77	R2	O-H	1.92
			Y99	R4	O-H	1.94
			Y99	R4	O-H	2.43

[a]Hydrogen bonding distance and binding energy were analyzed in Molsoft ICM-pro 3.5 computer program. Nine amino acids in each 9-mer epitope have been numbered 1-9.

Additional file 11-Figure S4: Docking of predicted strongest epitope 301-IDPFRLLQNSQVYSL-315 from IvAgNP with HLA-A2 (A), A3 (B), B27 (C), B39 (D) and B58 (E)[a]

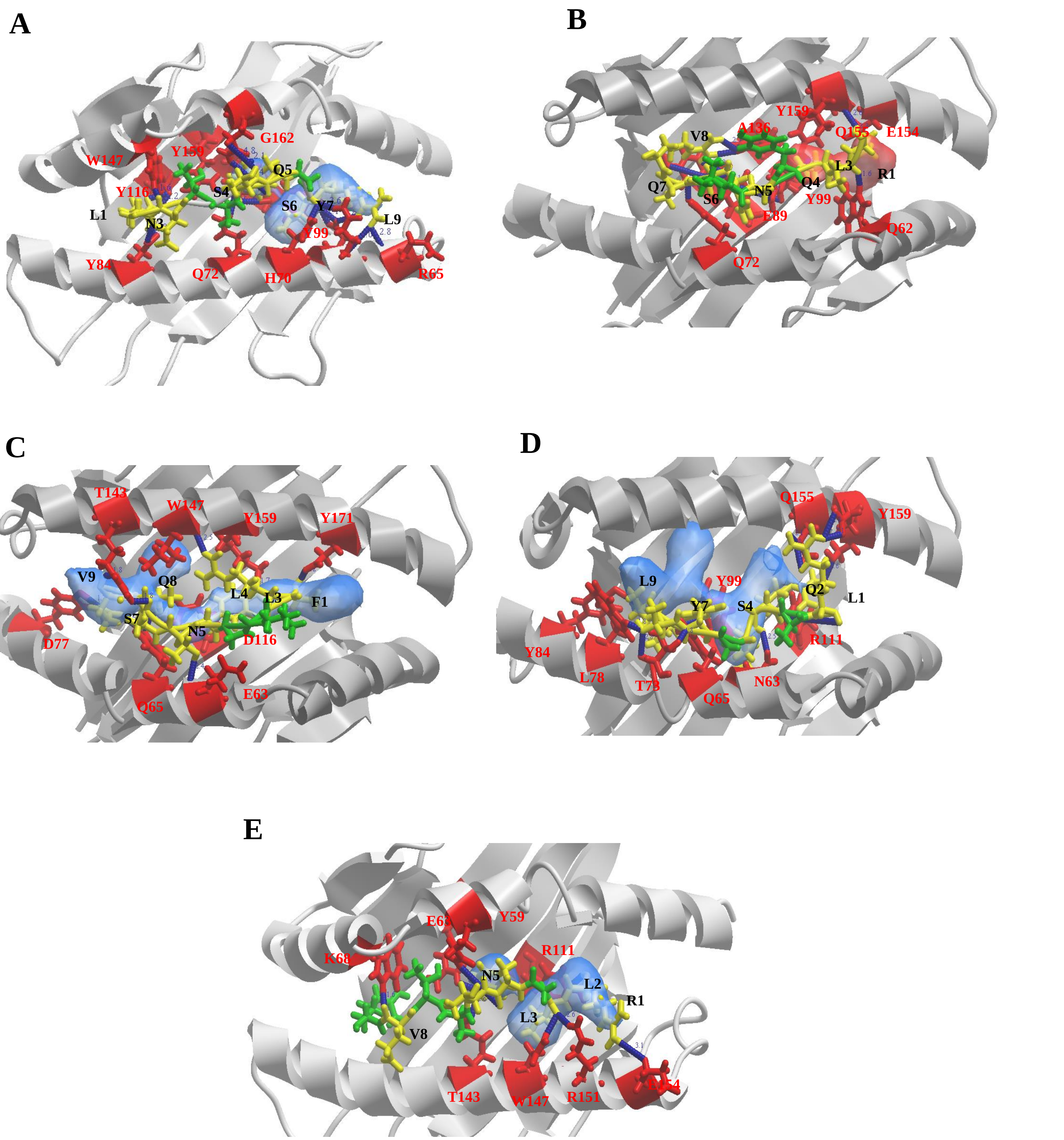

[a]MHC I structures are shown in grey ribbon and the amino acid residues involved in the H-bonding interaction are shown as red sticks and labeled red. The epitope is shown as in green ribbon and the amino acid residues involved in the H-bonding network are shown as yellow sticks and labeled black. MHC I binding pocket(s) are shown as electrostatic sphere. H-bonds are displayed as blue spheres and the H-bonding distances are labeled blue. H-bonding interaction between the amino acid residues of the epitope and those of the MHC I are detailed in additional file 12- Table S8

Additional file 12-Table S8: Hydrogen Bonds and binding statistics of predicted strongest epitope 301-IDPFRLLQNSQVYSL-315 from IvAgNP with MHC I complexes[a]

Epitope-MHC I complex	No. of H-bonds	Binding energy (kcal/mol)	Receptor residues in MHC I	Ligand residues in epitope	Interacting atoms	H-bond distance (Å)
Epitope (307-LQNSQVYSL-315)-HLA-A2	13	-195.63	Q72	S4	H-O	2.79
			Y116	L1	H-O	2.66
			W147	Q2	H-O	2.58
			Y159	S4	H-O	1.84
			Y159	S4	H-O	1.92
			R65	L9	O-H	2.70
			Y99	Y7	O-H	2.11
			Y159	S6	O-H	2.22
			G162	Y7	O-H	2.13
			Y84	N3	O-H	1.90
			R65	L9	O-H	2.35
			Y84	N3	H-O	2.40
			H70	Y7	H-O	3.00
Epitope (305-RLLQNSQVY-313)-HLA-A3	11	-204.35	Q62	R1	H-O	2.18
			Q72	N5	H-O	1.75
			Q72	N5	H-O	2.71
			Y159	L3	H-O	2.55
			E154	L3	H-O	1.49
			Y99	Q4	O-H	2.70
			A136	S6	O-H	1.80
			E89	Q7	H-O	1.68
			E89	Q7	H-O	1.91
			R108	V8	H-O	2.52
			Q155	V8	H-O	2.10
Epitope (304-FRLLQNSQV-312)-HLA-B27	11	-199.62	E63	N5	H-O	2.61
			Y159	L4	H-O	2.07
			Q65	N5	H-O	1.95
			D77	V9	H-O	1.83
			T143	V9	H-O	2.01
			W147	Q8	H-O	2.50
			Y159	L4	H-O	2.35
			Y171	F1	H-O	2.14
			E63	L9	O-H	2.40
			D116	L3	O-H	1.88
			D116	L3	O-H	2.41
Epitope (307-LQNSQVYSL-315)-HLA-B39	10	-191.13	R111	L1	H-O	1.81
			N63	S4	H-O	2.51
			Y99	Y7	H-O	2.31
			Y99	S4	H-O	2.39
			Y159	Q2	H-O	1.72
			Q155	Q2	O-H	1.73
			Q65	Y7	O-H	1.90
			T73	Y7	O-H	2.61
			Y84	L9	O-H	2.52
			L78	L9	O-H	2.66
			T73	Y7	O-H	2.67
Epitope (305-RLLQNSQVY-313)-HLA-B58	9	-185.32	E154	R1	H-O	3.11
			R151	L2	H-O	2.61
			W147	L3	H-O	2.64
			R111	L2	H-O	1.82
			T143	N5	O-H	1.63
			K68	V8	O-H	2.24
			E63	N5	O-H	2.31
			Y59	N5	H-O	1.70
			E63	N5	H-O	1.61

[a]Hydrogen bonding distance and binding energy were analyzed in Molsoft ICM-pro 3.5 computer program. Nine amino acids in each 9-mer epitope have been numbered 1-9.

Additional file 13-Figure S5: Multiple alignments of amino acid sequences of HLA-A and HLA-B series

```
                                                                            59                       84
HLA-A02   GSHSMRYFFTSVSRPGRGEPRFIAVGYVDDTQFVRFDSDAASQRMEPRAPWIEQEGPEYWDGETRKVKAHSQTHRVDLGTLRGYYNQSE 89
HLA-A24   GSHSMRYFSTSVSRPGRGEPRFIAVGYVDDTQFVRFDSDAASQRMEPRAPWIEQEGPEYWDEETGKVKAHSQTDRENLRIALRYYNQSE 89
HLA-A11   GSHSMRYFYTSVSRPGRGEPRFIAVGYVDDTQFVRFDSDAASQRMEPRAPWIEQEGPEYWDQETRNVKAQSQTDRVDLGTLRGYYNQSE 89
HLA-A25   GSHSMRYFYTSVSRPGRGEPRFIAVGYVDDTQFVRFDSDAASQRMEPRAPWIEQEGPEYWDRNTRNVKAHSQTDRESLRIALRYYNQSE 89
HLA-A36   GSHSMRYFFTSVSRPGRGEPRFIAVGYVDDTQFVRFDSDAASQKMEPRAPWIEQEGPEYWDQETRNMKAHSQTDRANLGTLRGYYNQSE 89
HLA-A33   GSHSMRYFTTSVSRPGRGEPRFIAVGYVDDTQFVRFDSDAASQRMEPRAPWIEQEGPEYWDRNTRNVKAHSQIDRVDLGTLRGYYNQSE 89
HLA-A32   GSHSMRYFFTSVSRPGRGEPRFIAVGYVDDTQFVRFDSDAASQRMEPRAPWIEQEGPEYWDQETRNVKAHSQTDRESLRIALRYYNQSE 89
HLA-A31   GSHSMRYFTTSVSRPGRGEPRFIAVGYVDDTQFVRFDSDAASQRMEPRAPWIEQERPEYWDQETRNVKAHSQIDRVDLGTLRGYYNQSE 89
HLA-A30   GSHSMRYFSTSVSRPGSGEPRFIAVGYVDDTQFVRFDSDAASQRMEPRAPWIEQERPEYWDQETRNVKAQSQTDRVDLGTLRGYYNQSE 89
HLA-A29   GSHSMRYFTTSVSRPGRGEPRFIAVGYVDDTQFVRFDSDAASQRMEPRAPWIEQEGPEYWDLQTRNVKAQSQTDRANLGTLRGYYNQSE 89
HLA-A74   GSHSMRYFFTSVSRPGRGEPRFIAVGYVDDTQFVRFDSDAASQRMEPRAPWIEQEGPEYWDQETRNVKAHSQTDRVDLGTLRGYYNQSE 89
HLA-A68   GSHSMRYFYTSVSRPGRGEPRFIAVGYVDDTQFVRFDSDAASQRMEPRAPWIEQEGPEYWDRNTRNVKAQSQTDRVDLGTLRGYYNQSE 89
HLA-A01   GSHSMRYFFTSVSRPGRGEPRFIAVGYVDDTQFVRFDSDAASQKMEPRAPWIEQEGPEYWDQETRNMKAHSQTDRANLGTLRGYYNQSE 89
HLA-A03   GSHSMRYFFTSVSRPGRGEPRFIAVGYVDDTQFVRFDSDAASQRMEPRAPWIEQEGPEYWDQETRNVKAQSQTDRVDLGTLRGYYNQSE 89
HLA-A34   GSHSMRYFYTSVSRPGRGEPRFIAVGYVDDTQFVRFDSDAASQRMEPRAPWIEQEGPEYWDRNTRKVKAQSQTDRVDLGTLRGYYNQSE 89
HLA-A26   GSHSMRYFYTSVSRPGRGEPRFIAVGYVDDTQFVRFDSDAASQRMEPRAPWIEQEGPEYWDRNTRNVKAHSQTDRANLGTLRGYYNQSE 89
HLA-B27   GSHSMRYFHTSVSRPGRGEPRFITVGYVDDTLFVRFDSDAASPREEPRAPWIEQEGPEYWDRETQICKAKAQTDREDLRTLLRYYNQSE 89
HLA-B44   GSHSMRYFYTAMSRPGRGEPRFITVGYVDDTLFVRFDSDATSPRKEPRAPWIEQEGPEYWDRETQISKTNTQTYRENLRTALRYYNQSE 89
HLA-B78   GSHSMRYFYTAMSRPGRGEPRFIAVGYVDDTQFVRFDSDAASPRTEPRAPWIEQEGPEYWDRNTQIFKTNTQTDRESLRNLRGYYNQSE 89
HLA-B67   GSHSMRYFYTSVSRPGRGEPRFISVGYVDDTQFVRFDSDAASPREEPRAPWIEQEGPEYWDRNTQIYKAQAQTDRESLRNLRGYYNQSE 89
HLA-B58   GSHSMRYFYTAMSRPGRGEPRFIAVGYVDDTQFVRFDSDAASPRTEPRAPWIEQEGPEYWDGETRNMKASAQTYRENLRIALRYYNQSE 89
HLA-B57   GSHSMRYFYTAMSRPGRGEPRFIAVGYVDDTQFVRFDSDAASPRMAPRAPWIEQEGPEYWDGETRNMKASAQTYRENLRIALRYYNQSE 89
HLA-B53   GSHSMRYFYTAMSRPGRGEPRFIAVGYVDDTQFVRFDSDAASPRTEPRAPWIEQEGPEYWDRNTQIFKTNTQTYRENLRIALRYYNQSE 89
HLA-B52   GSHSMRYFYTAMSRPGRGEPRFIAVGYVDDTQFVRFDSDAASPRTEPRAPWIEQEGPEYWDRETQISKTNTQTYRENLRIALRYYNQSE 89
HLA-B51   GSHSMRYFYTAMSRPGRGEPRFIAVGYVDDTQFVRFDSDAASPRTEPRAPWIEQEGPEYWDRNTQIFKTNTQTYRENLRIALRYYNQSE 89
HLA-B46   GSHSMRYFYTAMSRPGRGEPRFIAVGYVDDTQFVRFDSDAASPRMAPRAPWIEQEGPEYWDRETQKYKRQAQTDRVSLRNLRGYYNQSE 89
HLA-B41   GSHSMRYFHTAMSRPGRGEPRFITVGYVDDTLFVRFDSDATSPRKEPRAPWIEQEGPEYWDRETQISKTNTQTYRESLRNLRGYYNQSE 89
HLA-B38   GSHSMRYFYTSVSRPGRGEPRFISVGYVDDTQFVRFDSDAASPREEPRAPWIEQEGPEYWDRNTQICKTNTQTYRENLRIALRYYNQSE 89
HLA-B35   GSHSMRYFYTAMSRPGRGEPRFIAVGYVDDTQFVRFDSDAASPRTEPRAPWIEQEGPEYWDRNTQIFKTNTQTYRESLRNLRGYYNQSE 89
HLA-B18   GSHSMRYFHTSVSRPGRGEPRFISVGYVDGTQFVRFDSDAASPRTEPRAPWIEQEGPEYWDRNTQISKTNTQTYRESLRNLRGYYNQSE 89
HLA-B15   GSHSMRYFYTAMSRPGRGEPRFIAVGYVDDTQFVRFDSDAASPRMAPRAPWIEQEGPEYWDRETQISKTNTQTYRESLRNLRGYYNQSE 89
HLA-B13   GSHSMRYFYTAMSRPGRGEPRFITVGYVDDTQFVRFDSDATSPRMAPRAPWIEQEGPEYWDRETQISKTNTQTYRENLRTALRYYNQSE 89
HLA-B39   GSHSMRYFYTSVSRPGRGEPRFISVGYVDDTQFVRFDSDAASPREEPRAPWIEQEGPEYWDRNTQICKTNTQTDRESLRNLRGYYNQSE 89
HLA-B73   GSHSMRYFHTSVSRPGRGEPRFITVGYVDDTQFVRFDSDAASPREEPRAPWIEQEGPEYWDRNTQICKAKAQTDRVGLRNLRGYYNQSE 89
HLA-B08   GSHSMRYFDTAMSRPGRGEPRFISVGYVDDTQFVRFDSDAASPREEPRAPWIEQEGPEYWDRNTQIFKTNTQTDRESLRNLRGYYNQSE 89
HLA-B07   GSHSMRYFYTSVSRPGRGEPRFISVGYVDDTQFVRFDSDAASPREEPRAPWIEQEGPEYWDRNTQIYKAQAQTDRESLRNLRGYYNQSE 89
HLA-B40   GSHSMRYFHTSVSRPGRGEPRFITVGYVDDTLFVRFDSDATSPRKEPRAPWIEQEGPEYWDRETQISKTNTQTYRESLRNLRGYYNQSE 89
HLA-B54   GSHSMRYFYTAMSRPGRGEPRFIAVGYVDDTQFVRFDSDAASPRGEPRAPWVEQEGPEYWDRNTQIYKAQAQTDRESLRNLRGYYNQSE 89
HLA-B55   GSHSMRYFYTAMSRPGRGEPRFIAVGYVDDTQFVRFDSDAASPREEPRAPWIEQEGPEYWDRNTQIYKAQAQTDRESLRNLRGYYNQSE 89
HLA-B56   GSHSMRYFYTAMSRPGRGEPRFIAVGYVDDTQFVRFDSDAASPREEPRAPWIEQEGPEYWDRNTQIYKAQAQTDRESLRNLRGYYNQSE 89
HLA-B15   GSHSMRYFYTAMSRPGRGEPRFIAVGYVDDTQFVRFDSDAASPRMAPRAPWIEQEGPEYWDRETQISKTNTQTYRESLRNLRGYYNQSE 89
          ******** *::**** ******:*****.* ********:* :  *****:*** **** :*   *  :*  * .*      ******

                   99                                             143 147     155  159
HLA-A02   AGSHTVQRMYGCDVGSDWRFLRGYHQYAYDGKDYIALKEDLRSWTAADMAAQTTKHKWEAAHVAEQLRAYLEGTCVEWLRRYLENGKETL 179
HLA-A24   AGSHTLQMMFGCDVGSDGRFLRGYHQYAYDGKDYIALKEDLRSWTAADMAAQITKRKWEAAHVAEQQRAYLEGTCVDGLRRYLENGKETL 179
HLA-A11   DGSHTIQIMYGCDVGPDGRFLRGYRQDAYDGKDYIALNEDLRSWTAADMAAQITKRKWEAAHAAEQQRAYLEGRCVEWLRRYLENGKETL 179
HLA-A25   DGSHTIQRMYGCDVGPDGRFLRGYQQDAYDGKDYIALNEDLRSWTAADMAAQITQRKWETAHEAEQWRAYLEGRCVEWLRRYLENGKETL 179
HLA-A36   DGSHTIQIMYGCDVGPDGRFLRGYRQDAYDGKDYIALNEDLRSWTAADMAAQITKRKWEAVHAAEQRRVYLEGTCVEWLRRYLENGKETL 179
HLA-A33   AGSHTIQMMYGCDVGSDGRFLRGYQQDAYDGKDYIALNEDLRSWTAADMAAQITQRKWEAARVAEQLRAYLEGTCVEWLRRHLENGKETL 179
HLA-A32   AGSHTIQMMYGCDVGPDGRLLRGYQQDAYDGKDYIALNEDLRSWTAADMAAQITQRKWEAARVAEQLRAYLEGTCVEWLRRYLENGKETL 179
HLA-A31   AGSHTIQMMYGCDVGSDGRFLRGYQQDAYDGKDYIALNEDLRSWTAADMAAQITQRKWEAARVAEQLRAYLEGTCVEWLRRYLENGKETL 179
HLA-A30   AGSHTIQIMYGCDVGSDGRFLRGYEQHAYDGKDYIALNEDLRSWTAADMAAQITQRKWEAARWAEQLRAYLEGTCVEWLRRYLENGKETL 179
HLA-A29   AGSHTIQMMYGCHVGSDGRFLRGYRQDAYDGKDYIALNEDLRSWTAADMAAQITQRKWEAARVAEQLRAYLEGTCVEWLRRYLENGKETL 179
HLA-A74   AGSHTIQMMYGCDVGPDGRLLRGYQQDAYDGKDYIALNEDLRSWTAADMAAQITQRKWEAARVAEQLRAYLEGTCVEWLRRYLENGKETL 179
HLA-A68   AGSHTIQMMYGCDVGSDGRFLRGYRQDAYDGKDYIALKEDLRSWTAADMAAQTTKHKWEAAHVAEQWRAYLEGTCVEWLRRYLENGKETL 179
HLA-A01   DGSHTIQIMYGCDVGPDGRFLRGYRQDAYDGKDYIALNEDLRSWTAADMAAQITKRKWEAVHAAEQRRVYLEGRCVDGLRRYLENGKETL 179
HLA-A03   AGSHTIQIMYGCDVGSDGRFLRGYRQDAYDGKDYIALNEDLRSWTAADMAAQITKRKWEAAHEAEQLRAYLDGTCVEWLRRYLENGKETL 179
HLA-A34   DGSHTIQRMYGCDVGPDGRFLRGYQQDAYDGKDYIALNEDLRSWTAADMAAQITQRKWETAHEAEQWRAYLEGTCVEWLRRYLENGKETL 179
HLA-A26   DGSHTIQRMYGCDVGPDGRFLRGYQQDAYDGKDYIALNEDLRSWTAADMAAQITQRKWETAHEAEQWRAYLEGRCVEWLRRYLENGKETL 179
HLA-B27   AGSHTLQNMYGCDVGPDGRLLRGYHQDAYDGKDYIALNEDLSSWTAADTAAQITQRKWEAARVAEQLRAYLEGECVEWLRRYLENGKETL 179
HLA-B44   AGSHIIQRMYGCDVGPDGRLLRGYDQDAYDGKDYIALNEDLSSWTAADTAAQITQRKWEAARVAEQDRAYLEGLCVESLRRYLENGKETL 179
HLA-B78   AGSHTWQTMYGCDVGPDGRLLRGHNQYAYDGKDYIALNEDLSSWTAADTAAQITQRKWEAAREAEQLRAYLEGLCVEWLRRHLENGKETL 179
HLA-B67   AGSHTLQRMYGCDVGPDGRLLRGHNQFAYDGKDYIALNEDLSSWTAADTAAQITQRKWEAARVAEQLRTYLEGTCVEWLRRYLENGKETL 179
HLA-B58   AGSHIIQRMYGCDLGPDGRLLRGHDQSAYDGKDYIALNEDLSSWTAADTAAQITQRKWEAARVAEQLRAYLEGLCVEWLRRYLENGKETL 179
HLA-B57   AGSHIIQVMYGCDVGPDGRLLRGHDQSAYDGKDYIALNEDLSSWTAADTAAQITQRKWEAARVAEQLRAYLEGLCVEWLRRYLENGKETL 179
HLA-B53   AGSHIIQRMYGCDLGPDGRLLRGHDQSAYDGKDYIALNEDLSSWTAADTAAQITQRKWEAARVAEQLRAYLEGLCVEWLRRYLENGKETL 179
HLA-B52   AGSHTWQTMYGCDVGPDGRLLRGHNQYAYDGKDYIALNEDLSSWTAADTAAQITQRKWEAAREAEQLRAYLEGLCVEWLRRHLENGKETL 179
HLA-B51   AGSHTWQTMYGCDVGPDGRLLRGHNQYAYDGKDYIALNEDLSSWTAADTAAQITQRKWEAAREAEQLRAYLEGLCVEWLRRHLENGKETL 179
HLA-B46   AGSHTLQRMYGCDVGPDGRLLRGHDQSAYDGKDYIALNEDLSSWTAADTAAQITQRKWEAAREAEQWRAYLEGLCVEWLRRYLENGKETL 179
HLA-B41   AGSHTWQRMYGCDVGPDGRLLRGHNQYAYDGKDYIALNEDLRSWTAADTAAQITQRKWEAARVAEQDRAYLEGTCVEWLRRYLENGKDTL 179
HLA-B38   AGSHTLQRMYGCDVGPDGRLLRGHNQFAYDGKDYIALNEDLSSWTAADTAAQITQRKWEAARVAEQLRTYLEGTCVEWLRRYLENGKETL 179
HLA-B35   AGSHIIQRMYGCDLGPDGRLLRGHDQSAYDGKDYIALNEDLSSWTAADTAAQITQRKWEAARVAEQLRAYLEGLCVEWLRRYLENGKETL 179
HLA-B18   AGSHTLQRMYGCDVGPDGRLLRGHDQSAYDGKDYIALNEDLSSWTAADTAAQITQRKWEAARVAEQLRAYLEGTCVEWLRRHLENGKETL 179
HLA-B15   AGSHTLQRMYGCDVGPDGRLLRGHDQSAYDGKDYIALNEDLSSWTAADTAAQITQRKWEAAREAEQWRAYLEGLCVEWLRRYLENGKETL 179
HLA-B13   AGSHIIQRMYGCDLGPDGRLLRGHNQLAYDGKDYIALNEDLSSWTAADTAAQITQLKWEAARVAEQLRAYLEGECVEWLRRYLENGKETL 179
HLA-B39   AGSHTLQRMYGCDVGPDGRLLRGHNQFAYDGKDYIALNEDLSSWTAADTAAQITQRKWEAARVAEQLRTYLEGTCVEWLRRYLENGKETL 179
HLA-B73   DGSHTWQTMYGCDMGPDGRLLRGYNQFAYDGKDYIALNEDLRSWTAADTAAQITQRKWEAARVAEQLRAYLEGECVEWLRRHLENGKETL 179
HLA-B08   AGSHTLQSMYGCDVGPDGRLLRGHNQYAYDGKDYIALNEDLRSWTAADTAAQITQRKWEAARVAEQDRAYLEGTCVEWLRRYLENGKDTL 179
HLA-B07   AGSHTLQSMYGCDVGPDGRLLRGHDQYAYDGKDYIALNEDLRSWTAADTAAQITQRKWEAAREAEQRRAYLEGECVEWLRRYLENGKDKL 179
HLA-B40   AGSHTLQSMYGCDVGPDGRLLRGHNQYAYDGKDYIALNEDLRSWTAADTAAQITQRKWEAARVAEQLRAYLEGECVEWLRRYLENGKETL 179
HLA-B54   AGSHTWQTMYGCDLGPDGRLLRGHNQLAYDGKDYIALNEDLSSWTAADTAAQITQRKWEAARVAEQLRAYLEGTCVEWLRRYLENGKETL 179
HLA-B55   AGSHTWQTMYGCDLGPDGRLLRGHNQLAYDGKDYIALNEDLSSWTAADTAAQITQRKWEAAREAEQLRAYLEGTCVEWLRRYLENGKETL 179
HLA-B56   AGSHTWQTMYGCDLGPDGRLLRGHNQLAYDGKDYIALNEDLSSWTAADTAAQITQRKWEAARVAEQLRAYLEGLCVEWLRRYLENGKETL 179
HLA-B15   AGSHTLQRMYGCDVGPDGRLLRGHDQSAYDGKDYIALNEDLSSWTAADTAAQITQRKWEAAREAEQWRAYLEGLCVEWLRRYLENGKETL 179
           ***  * * **.:*.* *:***: * **********:*** ****** *** *: * *:.: *** *.**:* **: ***:*****:.*
```

The HLA-A02 sequence is chosen as reference. Position 59 (Y), 94 (Y), 99 (Y), 142 (T), 147 (W), 155 (Q) and 159 (Y) are marked as boxes to present no polymorphic binding site in the MHC protein. All Binding residues are labeled red and underlined.
